# TSP50 facilitates breast cancer stem cell-like properties maintenance and epithelial-mesenchymal transition via PI3K p110α mediated activation of AKT signaling pathway

**DOI:** 10.1186/s13046-024-03118-4

**Published:** 2024-07-20

**Authors:** Feng Gao, Sichen Liu, Jing Wang, Gang Wei, Chunlei Yu, Lihua Zheng, Luguo Sun, Guannan Wang, Ying Sun, Yongli Bao, Zhenbo Song

**Affiliations:** 1 National Engineering Laboratory for Druggable Gene and Protein Screening, Northeast Normal University, NO.5268 Renmin Street, Changchun, 130117 China; 2 NMPA Key Laboratory for Quality Control of Cell and Gene Therapy Medicine Products, Northeast Normal University, NO.5268 Renmin Street, Changchun, 130117 China; 3 China International Joint Research Center for Human Stem Cell Bank, Northeast Normal University, Changchun, Jilin, 130024 China; 4 Department of Neurosurgery/Neuro-Oncology, Sun Yat-Sen University Cancer Center, State Key Laboratory of Oncology in South China, Collaborative Innovation Center for Cancer Medicine, Guangzhou, Guangdong 510060 China; 5 Department of Breast Surgery, Jilin Province Cancer Hospital, Changchun, 130012 China

**Keywords:** Breast cancer, TSP50, CSC-like properties, EMT, PI3K/AKT pathway

## Abstract

**Background:**

Studies have confirmed that epithelial-mesenchymal transition (EMT) and cancer stem cell (CSC)-like properties are conducive to cancer metastasis. In recent years, testes-specific protease 50 (TSP50) has been identified as a prognostic factor and is involved in tumorigenesis regulation. However, the role and molecular mechanisms of TSP50 in EMT and CSC-like properties maintenance remain unclear.

**Methods:**

The expression and prognostic value of TSP50 in breast cancer were excavated from public databases and explored using bioinformatics analysis. Then the expression of TSP50 and related genes was further validated by quantitative RT-PCR (qRT-PCR), Western blot, and immunohistochemistry (IHC). In order to investigate the function of TSP50 in breast cancer, loss- and gain-of-function experiments were conducted, both in vitro and in vivo. Furthermore, immunofluorescence (IF) and immunoprecipitation (IP) assays were performed to explore the potential molecular mechanisms of TSP50. Finally, the correlation between the expression of TSP50 and related genes in breast cancer tissue microarray and clinicopathological characteristics was analyzed by IHC.

**Results:**

TSP50 was negatively correlated with the prognosis of patients with breast cancer. TSP50 promoted CSC-like traits and EMT in both breast cancer cells and mouse xenograft tumor tissues. Additionally, inhibition of PI3K/AKT partly reversed TSP50-induced activation of CSC-like properties, EMT and tumorigenesis. Mechanistically, TSP50 and PI3K p85α regulatory subunit could competitively interact with the PI3K p110α catalytic subunit to promote p110α enzymatic activity, thereby activating the PI3K/AKT signaling pathway for CSC-like phenotypes maintenance and EMT promotion. Moreover, IHC analysis of human breast cancer specimens revealed that TSP50 expression was positively correlated with p-AKT and ALDH1 protein levels. Notably, breast cancer clinicopathological characteristics, such as patient survival time, tumor size, Ki67, pathologic stage, N stage, estrogen receptor (ER) and progesterone receptor (PR) levels, correlated well with TSP50/p-AKT/ALDH1 expression status.

**Conclusion:**

The effects of TSP50 on EMT and CSC-like properties promotion were verified to be dependent on PI3K p110α. Together, our study revealed a novel mechanism by which TSP50 facilitates the progression of breast cancer, which can provide new insights into TSP50-based breast cancer treatment strategies.

**Supplementary Information:**

The online version contains supplementary material available at 10.1186/s13046-024-03118-4.

## Introduction

Breast cancer is one of the most prevalent malignancies among women worldwide and is a serious threat to their health [1, 2]. Chemotherapy, radiation and surgery are currently the most important and successful therapeutic modalities for breast cancer [3, 4]. However, when given a diagnosis of breast cancer, 20%-30% of patients already have distant metastases, which decreases their chance of survival by 74% [5]. Therefore, identifying the crucial biomarkers and comprehending the underlying factors of breast cancer recurrence may encourage the development of novel therapeutic and diagnostic approaches to improve the prognosis of breast cancer.

CSCs are a component of the cellular hierarchy and possess CSC-like properties, such as the ability to self-renew and potential for abnormal differentiation [6, 7]. As a key driver of malignant cancer progression, CSCs are closely associated with tumorigenesis, metastasis, chemoresistance and carcinoma recurrence, which may be a serious obstacle in the treatment of cancer [8, 9]. A protein which is homologous to serine proteases is encoded by a testis-specific gene, known as TSP50. As a proto-oncogene, TSP50 is inappropriately reactivated in a large number of malignant breast carcinoma tissues in addition to normal testes [10, 11]. TSP50 stimulates the growth of a variety of tumor cells [12–16], the effect of TSP50 on CSC-like properties, however, is not well understood. The complex biological process called EMT converts epithelial cells into cells with a mesenchymal phenotype [17]. Studies have shown that EMT has a direct impact on tumor aggressiveness, metastasis and stemness, in addition to embryonic development and wound healing [18]. TSP50 has been shown to facilitate gastric cancer invasion and metastasis by triggering EMT [19]. Similarly, the metastasis and invasion of breast cancer cells are also regulated by TSP50 [20]. TSP50’s potential impact on EMT in breast cancer remains largely unknown. Numerous studies have demonstrated that the AKT pathway is a key regulator of EMT and CSCs phenotype [21–24]. Nevertheless, TSP50’s effects on the AKT signaling pathway and a furthering influence on CSC-like properties and EMT in breast cancer have not been investigated.

In this study, we demonstrated that TSP50 activates the PI3K/AKT pathway through interaction with PI3K p110α, thereby enhancing CSC-like phenotypes and EMT in breast cancer cells. Our results support the potentially important role of TSP50 in CSC-like phenotypes maintenance and EMT of human breast cancer, which providing new insights into the treatment of breast cancer by targeting TSP50.

## Materials and methods

### Key resources

The reagents and bioinformatics analysis tools required for the study were listed in Additional file 1: Key Resources Table.

### Clinical correlation analysis of TSP50 expression with breast cancer

TISIDB (http://cis.hku.hk/TISIDB/index.php) [25], an integrated repository portal for tumor-immune system interactions, was used to analyze the expression of TSP50 in different molecular subtypes of breast cancer. Furthermore, we evaluated the prognostic significance of TSP50 in KM plotter online database (http://kmplot.com/) [26], patients were split by auto select best cutoff. The ROC of therapy-related survival and differences in TSP50 expression between responders and non-responders were investigated using the ROC plotter (www.rocplot.org), a web-based tool that connects gene expression to therapeutic response [27]. In addition, the GEPIA2 database (http://gepia2.cancer-pku.cn/#index) [28] was used to perform expression correlation analysis of TSP50 with breast cancer stem cell (BCSC) markers, including OCT4, ALDH1, NANOG and CD44.

### Cell culture, drug treatment and plasmid transfection

The human breast cancer cell lines MDA-MB-231, MCF7, T47D, ER7530 and SKBR3 were obtained from the Chinese Academy of Sciences and cultured in RPMI 1640 medium with 10% FBS, 100 units/mL penicillin and 100 mg/mL streptomycin. All the cells were maintained at 37 °C in an incubator with 5% CO2. For drug treatment, breast cancer cells were incubated with 10 µM SC79, 20 µM LY294002 or 5 µM BYL-719 for subsequent experiments. For transfection, 200 µL of RPMI 1640 serum-free medium was mixed with 6 µL of X-tremeGENE HP and 2 µg of plasmid when the cell confluence reached 80%. The cells were harvested for subsequent experiments after the specified transfection time.

### Lentiviral-mediated stable transfection of breast cancer cells

We utilized lentiviral vectors obtained from Miaoling Bio., which encode control, shTSP50 for TSP50 knockdown, and TSP50 overexpression sequences, to establish stable transfection in breast cancer cells. The shRNA sequence designed to target TSP50 was as follows: GGAACTCAAGTACAGCAATTATTCAAGAGATAATTGCTGTACTTGAGTTCCTT. For the transfection process, breast cancer cells were plated in a 24-wells plate at a density of 5 × 10^4^ cells per well and incubated for 24 h. Subsequently, the cells were infected with the lentiviral particles for an additional 24 h. To enhance the infection efficiency, polybrene was added according to the manufacturer’s protocol. Finally, stably transfected cell clones were established by the addition of puromycin.

### Mammosphere formation and analysis

Breast cancer cells were digested into single cells and subsequently seeded in ultralow attachment plates at a density of 500 or 3000 cells per well. The cells were cultured in epidermal growth factor (EGF) and basic fibroblast growth factor (bFGF) added DMEM/F12 serum-free culture medium for 10–14 days. The number and diameter of formed mammospheres were then quantified. For the assessment of self-renewal capacity, the mammospheres were collected, dissociated into single cells, and then reseeded under the same culture conditions as described above for secondary sphere formation.

### Extreme limiting dilution analysis (ELDA) for mammosphere formation

Breast cancer cells were seeded in 96-well ultralow attachment plates at densities of 2, 4, 8, 16, 32 and 64 cells per well for the limited dilution experiment. The number of mammospheres formed in each well was counted after 10 days. To quantitatively analyze the results of the limited dilution assay, we employed ELDA (http://bioinf.wehi.edu.au/software/elda) to determine the mammosphere synthesis efficiency [29].

### qRT-PCR detection and Western blot

Total RNA of cells or tissues was extracted using Trizol reagent and reverse transcribed into cDNA using a reverse transcription kit after the integrity was confirmed by electrophoresis. A SYBR Green kit was then employed for qRT-PCR experiment according to the manufacturer’s instructions. The specific primers were designed and listed in Table 1.

**Table 1:** Primer sequences for qRT-PCR detection

*Symbol*	*Primer*	*Primer Sequence (5′–3′)*
TSP50	F-Primer	ACAGGGAGGAGTTCTGCTATGAGATAAC
	R-Primer	AAAGATGGGTGGGGCCTCGCTCTTCTTG
NANOG	F-Primer	CCTGTGATTTGTGGGCCTGA
	R-Primer	CTCTGCAGAAGTGGGTTGTTTG
OCT4	F-Primer	GAGAACCGAGTGAGAGGCAACC
	R-Primer	CATAGTCGCTGCTTGATCGCTTG
ALDH1	F-Primer	GGGCTGTTCGAGAGGTTCG
	R-Primer	CAGGGCAAATCCTCCACATCA
CD44	F-Primer	AAGGAGAATACAGAACGAA
	R-Primer	AGAAACAACCATGAAAACC
E-cad	F-Primer	ATTTTTCCCTCGACACCCGAT
	R-Primer	TCCCAGGCGTAGACCAAGA
MMP9	F-Primer	AGACCTGGGCAGATTCCAAAC
	R-Primer	CGGCAAGTCTTCCGAGTAGT
Slug	F-Primer	TGTGACAAGGAATATGTGAGCC
	R-Primer	TGAGCCCTCAGATTTGACCTG
Snail	F-Primer	ACTGCAACAAGGAATACCTCAG
	R-Primer	GCACTGGTACTTCTTGACATCTG
GAPDH	F-Primer	ACAACTTTGGTATCGTGGAAGG
	R-Primer	GCCATCACGCCACAGTTTC

RIPA buffer was used to extract the total protein from the cells or tissues, which was then separated by SDS-PAGE and transferred to a PVDF membrane. Nonspecific antigen sites were inhibited with 5% skim milk and the membranes were incubated with primary antibodies at the suggested dilutions. Finally, ECL luminous fluid was used to track the protein signals. Gray values of the proteins were analyzed using Image J software and normalized to GAPDH.

### IP, Co-immunoprecipitation (Co-IP) and IF

The protein A/G magnetic beads were used for IP and Co-IP detection. The IP, Co-IP and IF were performed with the specific primary antibodies (IP and Co-IP experiments: anti-TSP50, anti-p110α and anti-flag. IF experiment: anti-TSP50 1:1000, anti-p110α 1:1000, anti-β-catenin 1:1000, anti-NICD 1:1000) as described previously [12].

### PI3K p110α activity assay

The total protein of the transfected breast cancer cells was extracted, and PI3K p110α was purified using the IP method. The activity of PI3K p110α was determined using a human PI3K elisa kit according to the manufacturer’s instructions.

### Aldehyde dehydrogenase (ALDH) activity assay

The cells were placed on ice, followed by the detection of ALDH using an ALDH test kit, according to the manufacturer’s instructions. The ALDH activity was evaluated at 340 nm using a microplate reader by measuring NAD + production. Stronger activity was indicated by higher optical density (OD) value.

### Flow cytometry

The transfected breast cancer cells were dispersed into individual cells and incubated with anti-CD44-PE and anti-CD24-APC antibodies for 30 min in the dark. The number of CD44^+^/CD24^−^ cells was analyzed by flow cytometry. The flow cytometry was also used for intracellular ADR pumping rate detection, as previously reported [30].

### Colony formation

The transfected breast cancer cells (200 cells/well) were seeded in 6-well plates and cultured for 14 days. The medium was refreshed every 3 days. The formed colonies were fixed with 4% paraformaldehyde for 15 min, and subsequently, stained with 0.1% crystal violet for 15 min. After abstersion with PBS, the colonies were photographed. Furthermore, the crystal violet was completely eluted by 33% glacial acetic acid. The eluent OD value was measured at 570 nm using a microplate reader, which represents the clone-forming ability.

### Wound healing

A pipette tip was used to generate a scratch in the monolayer of transfected breast cancer cells (approximately 100% confluence) for the wound healing assay. The adherent cells were then gently washed twice with PBS to wipe out the floating cells and cultured in serum-free medium. The migration distance of breast cancer cells at 0 and 48 h was observed using a microscope, and the wound closure rate was then evaluated.

### Transwell invasion and migration

To measure cell invasive and migratory capabilities, the transwell chambers were pre-coated with or without Matrigel. The membrane of upper chamber was infiltrated with serum-free medium and plated with the transfected breast cancer cells. Meanwhile, the bottom of the chamber was supplemented with FBS-added culture medium. At 48 h post-incubation, the cells that crossed the membranes into the lower chamber were fixed with 4% paraformaldehyde for 15 min, and subsequently, stained with 0.1% crystal violet for 15 min. An inverted microscope was used to photograph the invasive and migratory cells. Finally, the crystal violet was completely eluted with 33% glacial acetic acid. The eluent OD value was measured at 570 nm using a microplate reader, which represented the number of invaded and migrated cells.

### Mouse xenograft and drug treatment assays

For serial dilution assays, lentivirus-infected experimental group and control group mammospheres were digested with trypsin (2 × 10^3^–2 × 10^6^ cells) and injected subcutaneously into both flanks (MDA-MB-231 cells, left flank for shNC group and right flank for shTSP50 group) and fat pads (MCF7 cells, left fat pad for LV-Con group and right fat pad for LV-TSP50 group) of 6-week-old female BALB/C nude mice (*n* = 2 in 2 × 10^6^ cell groups, *n* = 6 in other groups), respectively. To analyze the effects of drugs on tumorigenesis, lentivirus-infected mammospheres were injected subcutaneously into both flanks (MDA-MB-231 cells, 1 × 10^6^ cells, left flank for shNC group and right flank for shTSP50 group) and fat pads (MCF7 cells, 1 × 10^6^ cells, left fat pad for LV-Con group and right fat pad for LV-TSP50 group) of the nude mice (*n* = 6 in each group), respectively. SC79 was administered at 0.04 mg/g/d (i.p.) [31], LY294002 at 0.05 mg/g (i.p.), twice weekly [32], and BYL-719 at 50 mg/kg/d (i.g.) [33], after the injection of tumor cells. The tumors were weighed using an electronic balance and a vernier caliper was used for tumor size measurement. Furthermore, the volumes were estimated using the equation (a^2^ × b)/2 (a, short diameter; b, long diameter). All animal studies were approved by the Animal Research Ethics Committee of Northeast Normal University (NENU/IACUC, AP20230315) of China and performed in accordance with the established guidelines.

### Lung metastasis model assay

A total of twenty 4-week-old nude mice were randomly divided into four groups (*n* = 5 in each group), and 2 × 10^6^ transfected MDA-MB-231 cells were collected and incubated with the Did solution for 15 min at 37 ℃ in the dark. The cells were then tail vein injected into the nude mice. Lung metastasis was measured by an in vivo imaging system after 4 weeks of treatment with 0.04 mg/g/d (i.p.) SC79. Lung tissues were collected for Haematoxylin–eosin (HE) staining.

### IHC staining

Nude mouse tumor tissues were fixed, then paraffin embedded and sectioned into slices (5 μm). Tissue microarrays (HBreD136Su02) were obtained from Outdo BioTech (Shanghai, China), containing 136 cases of breast cancer specimens with the clinical data of patient survival, age, tumor volume, Ki67 level, axillary lymph node metastasis, pathologic stage, TNM stage, ER level, PR level and HER-2 level. After dewaxing and rehydration, IHC was performed as previously described [20].

### Statistical analysis

A log-rank test was used to assess the survival significance. The correlations were quantified using Pearson’s correlation coefficients. Statistical evaluation was performed using IBM SPSS Statistics Software. Data from three independent experiments were presented as mean ± S.D. Two-sided Student’s t-test and one-way ANOVA test were used for statistical analysis. In addition, the correlation of TSP50 with p-AKT and ALDH1, and the association between TSP50/p-AKT/ALDH1 and important clinical indicators were analyzed by Spearman and Chi-square tests. Statistical significance was indicated by *p < *0.05* and **p < *0.01*, ns, no significance.

## Results

### TSP50 is expressed differently in distinct breast cancer molecular subtypes and is associated with therapeutic responses, survival and BCSC markers

**Fig. 1:**
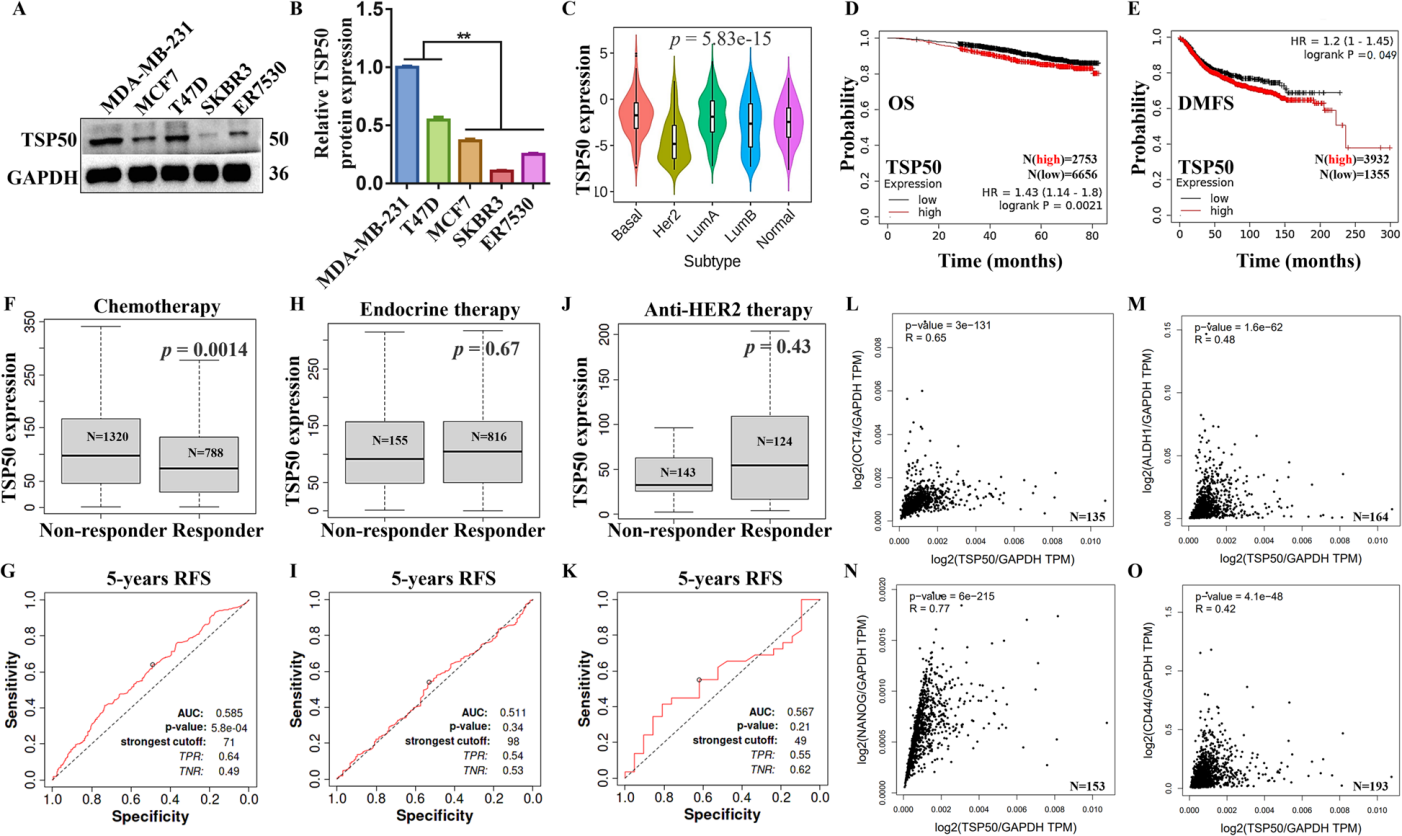
TSP50 is expressed differently in distinct breast cancer molecular subtypes and is associated with therapeutic responses, survival and BCSC markers. **A**, **B** Western blot results of TSP50 expression levels in different breast cancer cells. **C** TISIDB analysis results of TSP50 expression in distinct molecular subtypes of breast cancer. **D**, **E** KM plotter analysis results of the correlation between TSP50 mRNA expression levels and OS or DMFS in breast cancer patients. **F-K** ROC plotter analysis results of TSP50 expression in responders and non-responders (**F**, **H** and **J**) and the predictive accuracy of patient therapeutic response by TSP50 levels (**G**, **I** and **K**). **L-O** The association between TSP50 expression and CSCs markers in breast cancer patients from the TCGA database. A log-rank test was used to assess the survival significance. Correlations were quantified using Pearson’s correlation coefficient. The Student’s t-test was used to estimate the significance of differences between two groups, and more than two groups were analyzed using one-way ANOVA. *N* = 3 biologically independent replicates for Western blot. **p < *0.01*

Firstly, we determined the expression levels of TSP50 in different breast cancer cells. Western blot results showed that TSP50 was highly expressed in MDA-MB-231 and T47D cells, compared with MCF7, SKBR3 and ER7530 cells (Fig. 1A and B). Therefore, higher-TSP50-expressed MDA-MB-231 and T47D cells and lower-TSP50-expressed MCF7 and ER7530 cells were selected for subsequent experiments. Meanwhile, we explored whether TSP50 was differentially expressed in distinct breast cancer molecular subtypes using TISIDB. The violin diagram exhibited that there were significant differences in the mRNA expression of TSP50 in basal, her2, lumA, lumB and normal subtypes (Fig. 1C). Then, a KM plotter was employed to generate survival curves, including overall survival (OS) and distant metastasis-free survival (DMFS), to assess the association between TSP50 expression and survival outcomes in breast cancer cohorts. The breast cancer patients were separated into two groups according to TSP50 mRNA expression levels. Finally, we found that the high TSP50 expression group had a shorter OS and DMFS (Fig. 1D and E). Thus, TSP50 could be served as a potential diagnostic indicator for breast cancer. To seek whether TSP50 can predict therapeutic responses to breast cancer, we obtained the data from a receiver operating characteristic (ROC) curves plotter to show the association between therapeutic outcomes and TSP50 expression in breast cancer. The results showed that the non-responders to chemotherapy harbored higher TSP50 expression, however, the AUC value of the post-chemotherapy 5-year progression-free survival (PFS) prediction just reached 0.585 (Fig. 1F and G). In addition, there was no significant difference in TSP50 expression between non-responders and responders after endocrine and anti-HER2 therapies. The AUC values of the 5-year PFS prediction after treatment were 0.511 and 0.567, respectively (Fig. 1H-K). According to TCGA data for breast cancer, TSP50 mRNA expression was positively correlated with OCT4, ALDH1, NANOG and CD44 markers associated with BCSC (Fig. 1L-O).

### Overexpression of TSP50 increases CSC-like phenotypes in breast cancer cells

**Fig. 2:**
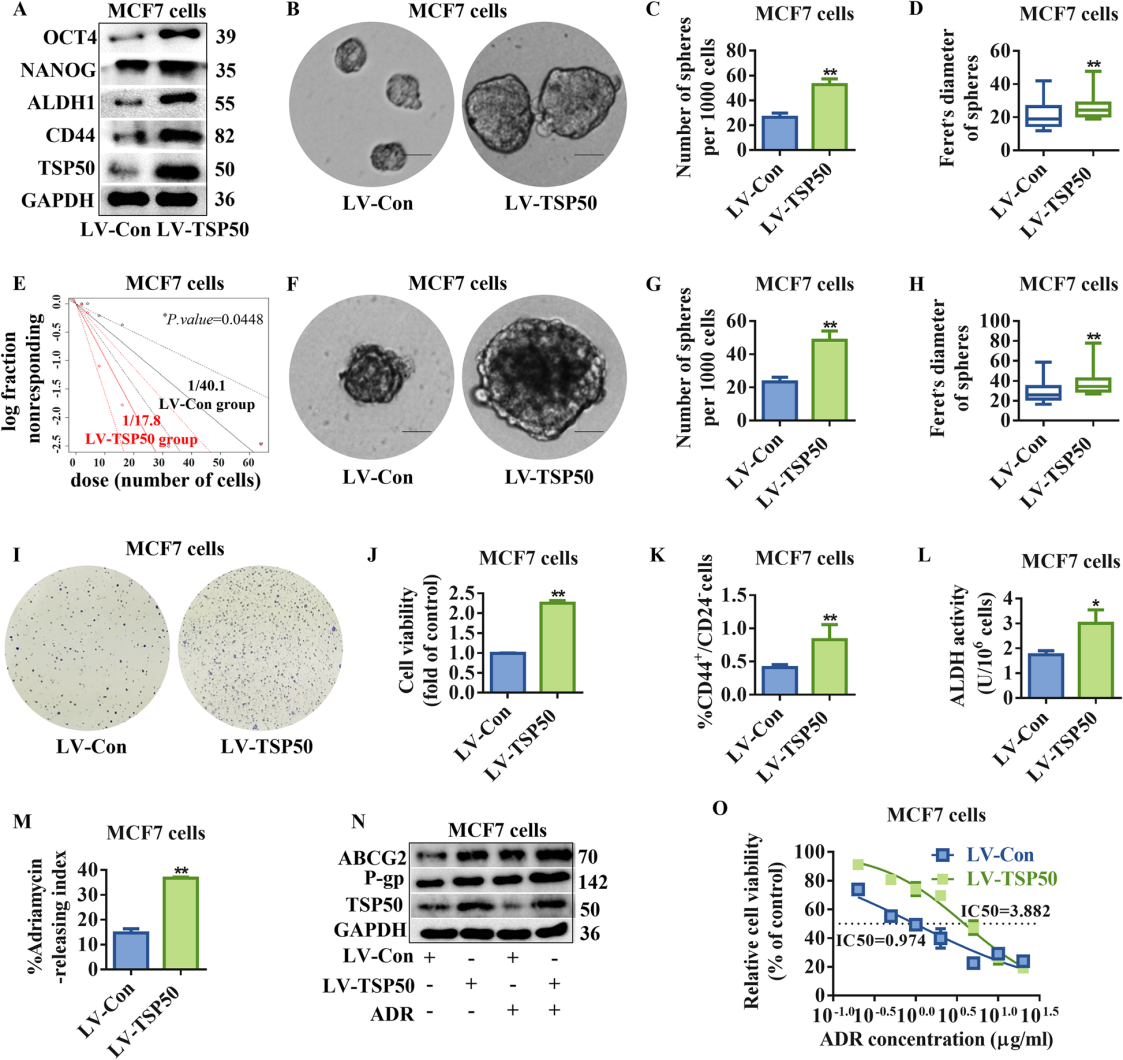
Overexpression of TSP50 increases CSC-like phenotypes in MCF7 cells. **A** The protein expression levels of BCSC-related markers OCT4, NANOG, ALDH1 and CD44 in MCF7 cells stably overexpressing NC or TSP50. **B** Representative mammosphere images derived from the NC or TSP50-overexpressed MCF7 cells. Scale bar, 25 μm. **C** Number of primary mammospheres. **D** Size of primary mammospheres. **E** The linear regression plot generated by ELDA for in vitro limiting dilution assay with NC or TSP50 transfected MCF7 cells. **F** Representative mammosphere images derived from the NC or TSP50-overexpressed mammospheres. Scale bar, 25 μm. **G** Number of secondary mammospheres. **H** Size of secondary mammospheres. **I**, **J** Representative colony formation images derived from the stable TSP50 or NC overexpressed MCF7 cells and the number of colonies analysis results. **K** The CD44^+^/CD24^−^ cell subpopulation proportion analysis results by flow cytometry. **L** ALDH activity detection results. **M**–**O** The ADR pumping rate (**M**), expression levels of drug resistance proteins ABCG2 and P-gp (**N**) and IC50 values of ADR (**O**) detection results in MCF7 cells stably overexpressing NC or TSP50. *N* = 3 biologically independent replicates. ^*^p < *0.05* and **p < *0.01*, as compared with control group by Student’s t-test

We created stable TSP50 or negative control (NC) overexpressed MCF7 and ER7530 cells using lentivirus to evaluate the role of TSP50 in the regulation of CSC-like properties. Notably, when compared to NC cells, TSP50 overexpression dramatically increased the mRNA and protein expression levels of the BCSC-related markers OCT4, NANOG, ALDH1 and CD44 (Fig. 2A, Fig. S1A, Fig. S2A and B). Sphere-forming experiments showed that TSP50 overexpression significantly increased the number and diameter of spherical cells, as well as the frequency of sphere-forming cells, as detected by an in vitro limiting dilution assay (Fig. 2B-E, Fig. S2C-F). Self-renewal was then measured according to the secondary mammosphere formation assay and colony formation experiments. Consistent with our hypothesis, TSP50 significantly promoted secondary mammosphere formation, and colony formation experiments revealed a much higher number of colonies in TSP50 overexpressed breast cancer cells (Fig. 2F-J, Fig. S2G-K). Additionally, BCSCs are characterized by important cell-surface and functional markers, such as CD44^+^/CD24^−/low^ and high ALDH activity [34, 35]. In this study, the proportion of CD44^+^/CD24^−^ cells and ALDH activity in the TSP50 overexpressed cells were markedly increased compared with the control (Fig. 2K and L, Fig. S2L-N). Adriamycin (ADR) is a common chemotherapy drug for breast cancer, however, it is more likely to develop drug resistance during clinical treatment due to the presence of CSCs [36]. Also, ABCG2 and P-gp have been shown to be frequently expressed in human cancer and act as xenobiotics efflux pumps to transport various classical chemotherapeutic agents out of cells [37]. Interestingly, we found that TSP50 overexpression increased the ADR pumping rate and the expression levels of drug resistance proteins ABCG2 and P-gp (Fig. 2M and N, Fig. S2O and P). And the sensitivity of breast cancer cells to ADR was reduced (Fig. 2O and Fig. S2Q).

**Fig. 3:**
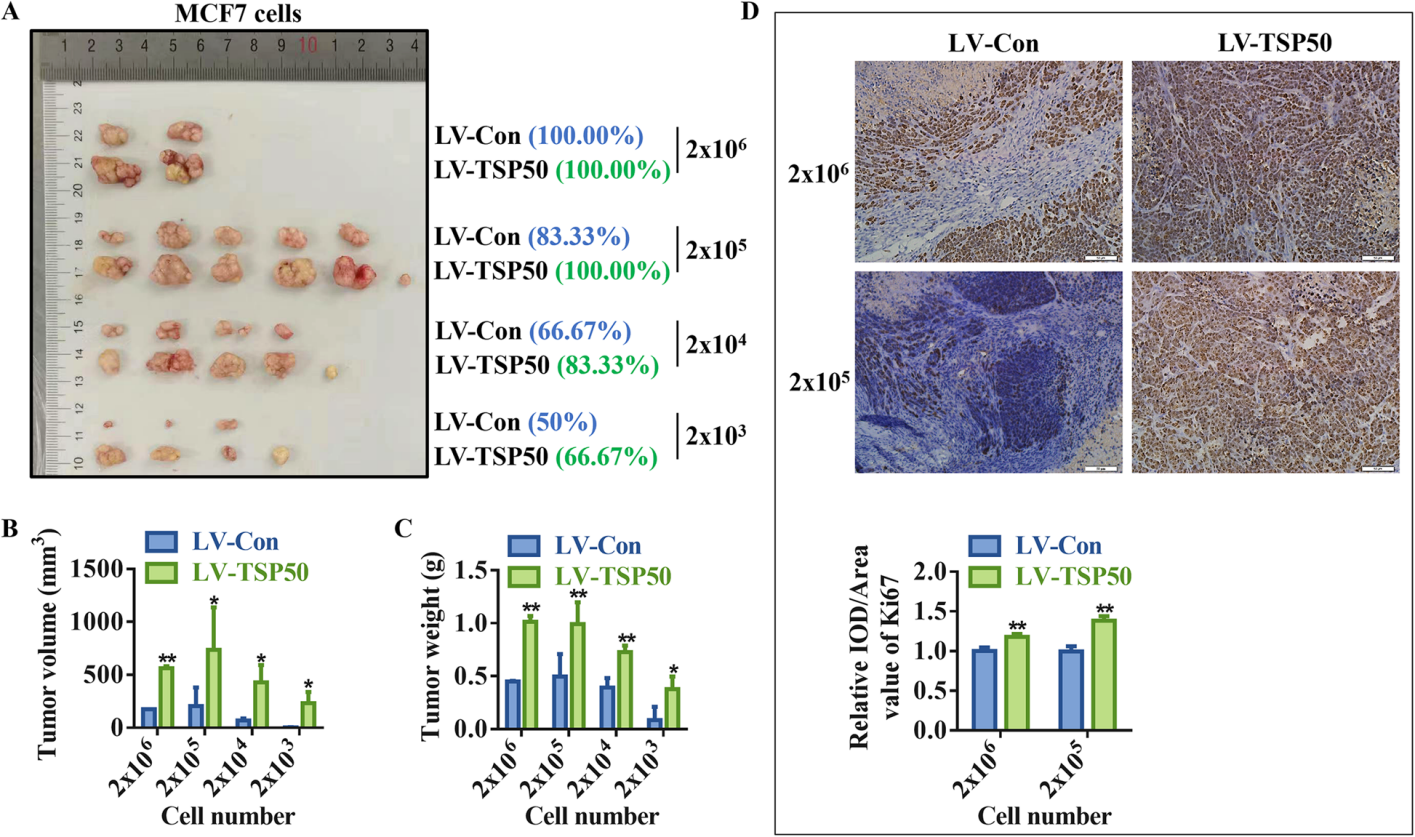
Overexpression of TSP50 promotes limited dilution tumorigenesis. **A-C** Stable overexpression of TSP50 or NC mammospheres were seeded into nude mice. The incidence of tumors was listed according to the number of seeded cells (**A**). Tumor volume (**B**) and weight (**C**) of each group. **D** Ki67 IHC analysis results in control or TSP50 overexpressed tumor tissues of nude mice. Scale bar, 50 μm. *N* = 3 biologically independent replicates for IHC assay (2 × 10^5^ cell groups). *N* = 2 biologically independent replicates for IHC assay (2 × 10^6^ cell groups). ^*^p < *0.05* and **p < *0.01*, as compared with the NC group by Student’s t-test

The limited dilution and tumorigenesis assay in vivo serves as the gold standard for CSCs testing. As shown in Fig. 3A-C, experiments using in vivo xenografts revealed that breast cancer cells overexpressing TSP50 had a considerably greater tumor-forming efficiency and a significantly larger tumor volume and weight than control cells. The qRT-PCR analysis results of tumor tissue lysates showed that the overexpression of TSP50 promoted the mRNA expression of BCSC-related markers NANOG, OCT4 and ALDH1 (Fig. S1B-E). Further IHC staining results proved that overexpressed TSP50 induced Ki67 expression in tumor tissues (Fig. 3D). These findings imply that the CSC-like properties of breast cancer cells are enhanced by TSP50.

### Inhibition of TSP50 attenuates CSC-like phenotypes in breast cancer cells

**Fig. 4:**
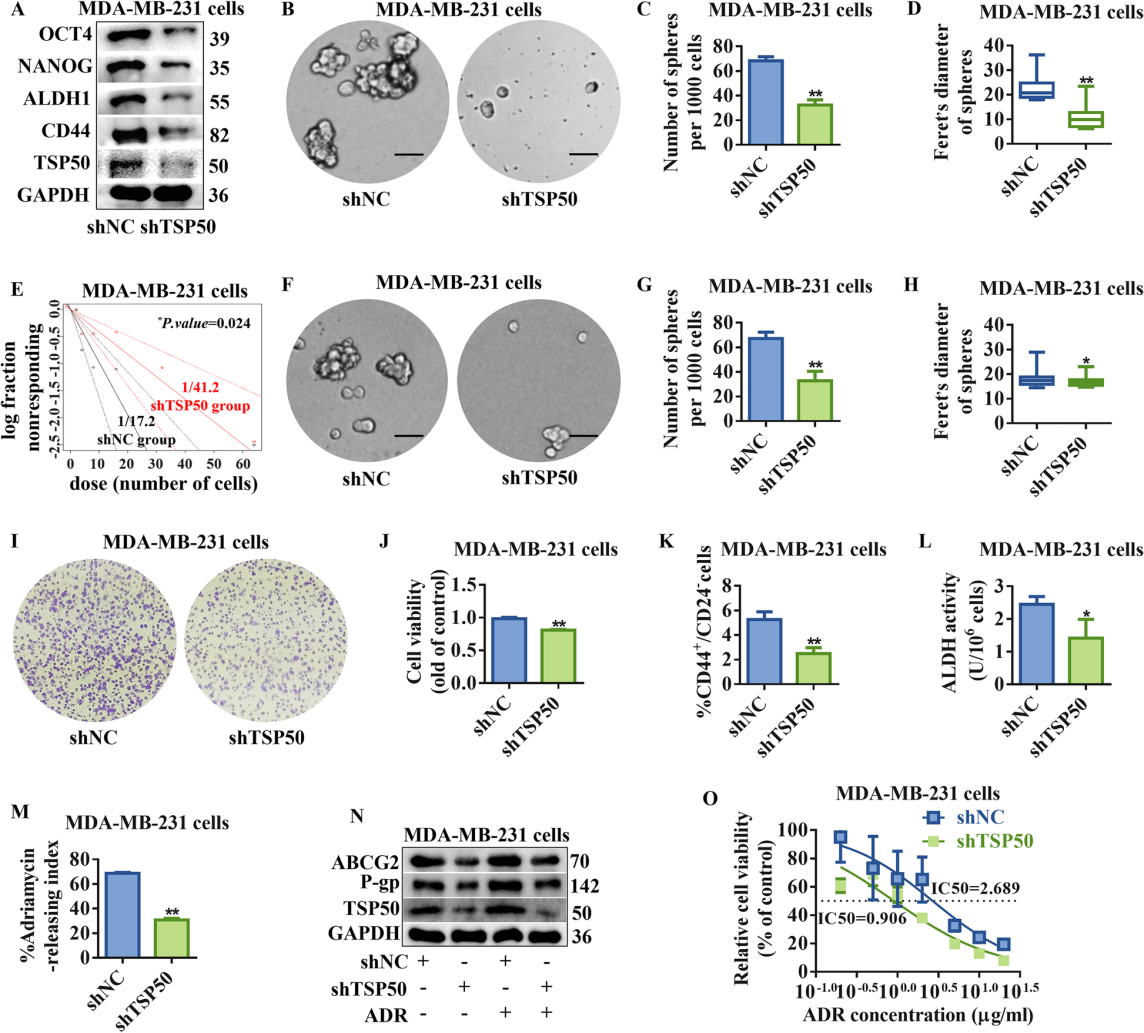
Inhibition of TSP50 attenuates CSC-like phenotypes in MDA-MB-231 cells. **A** The protein expression levels of BCSC-related markers OCT4, NANOG, ALDH1 and CD44 in MDA-MB-231 cells transfected with TSP50 shRNA (shTSP50) and control (shNC). **B** Representative mammosphere images of MDA-MB-231 cells transfected with shNC or shTSP50. Scale bar, 25 μm. **C** Primary mammospheres number. **D** The size of primary mammospheres. **E** The linear regression plot generated by ELDA for in vitro limiting dilution assay with MDA-MB-231-shNC or MDA-MB-231-shTSP50 cells. **F** Representative mammosphere images derived from the mammospheres transfected with shNC or shTSP50. Scale bar, 25 μm. **G** Secondary mammospheres number. **H** The size of secondary mammospheres. **I**, **J** Representative colony formation images derived from the shNC or shTSP50 transfected MDA-MB-231 cells and the number of colonies analysis results. **K** The CD44^+^/CD24^−^ cells subpopulation proportion analysis results by flow cytometry. **L** ALDH activity detection results. **M**–**O** The ADR pumping rate (**M**), expression levels of drug resistance proteins ABCG2 and P-gp (**N**) and IC50 values of ADR (**O**) detection results in shNC or shTSP50 transfected MDA-MB-231 cells. *N* = 3 biologically independent replicates. ^*^p < *0.05* and **p < *0.01*, as compared with control group by Student’s t-test

**Fig. 5:**
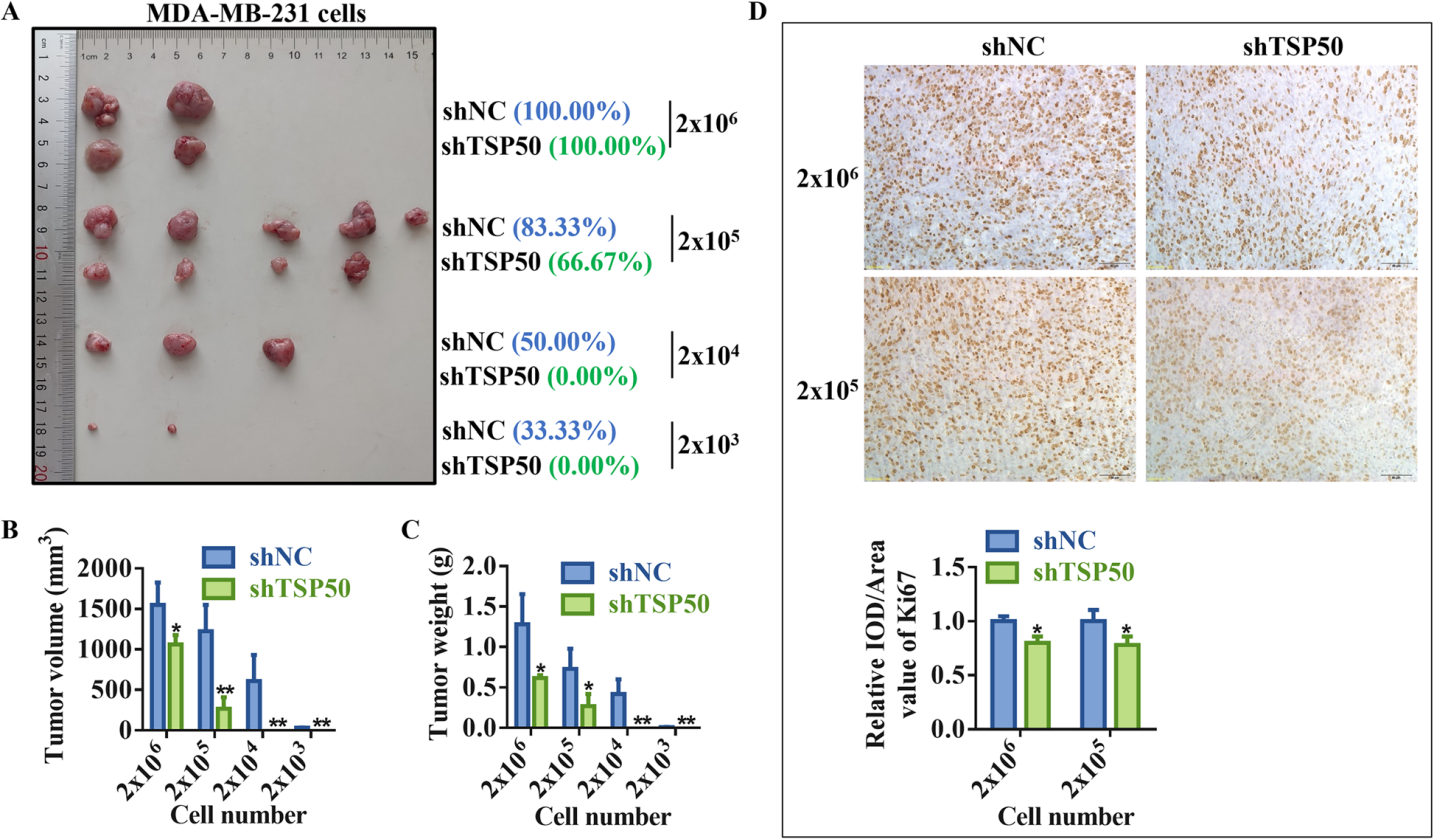
TSP50 knockdown inhibits limited dilution tumorigenesis. **A-C** Stable TSP50-knockdown and shNC mammospheres were seeded into nude mice. The incidence of tumors was listed according to the seeded cell number (**A**). Tumor volume (**B**) and tumor weight (**C**) for each group. **D** Ki67 IHC analysis results in control or TSP50 knockdown tumor tissues of nude mice. Scale bar, 50 μm. *N* = 3 biologically independent replicates for IHC assay (2 × 10^5^ cell groups). *N* = 2 biologically independent replicates for IHC assay (2 × 10^6^ cell groups). ^*^p < *0.05* and **p < *0.01*, as compared with shNC group by Student’s t-test

To further establish TSP50’s role in CSC-like properties maintenance of breast cancer cells, a lentivirus vector expressing shRNA for TSP50 knockdown was developed. Following TSP50 suppression, the expression of stemness genes was markedly downregulated in MDA-MB-231 and T47D cells (Fig. 4A, Fig. S1F, Fig. S3A and B). Smaller and fewer mammospheres were produced as a result of TSP50 suppression during primary and secondary mammosphere formation (Fig. 4B-H, Fig. S3C-I). Additionally, TSP50 knockdown breast cancer cells formed significantly fewer colonies than cells carrying an empty vector, as demonstrated by the colony formation assay (Fig. 4I and J, Fig. S3J and K). Meanwhile, TSP50 shRNA-stably transfected breast cancer cells revealed a lower CD44^+^/CD24^−^ cell ratio and ALDH activity (Fig. 4K and L, Fig. S3L-N). Furthermore, lower expression of TSP50 also decreased the ADR pumping rate, drug resistance protein levels, and the sensitivity of breast cancer cells to ADR was enhanced (Fig. 4M-O, Fig. S3O-Q). The subsequent in vivo results indicated that breast cancer cells with decreased expression of TSP50 had a lower tumor-forming efficiency, besides tumor volume and weight (Fig. 5A-C). Meanwhile, NANOG, OCT4 and ALDH1 mRNA and Ki67 protein levels were dropped in tumor tissues of the TSP50 knockdown group (Fig. 5D, Fig. S1G-J). These results suggest that the inhibition of TSP50 attenuates breast cancer CSC-like properties.

### TSP50 promotes breast cancer cell metastasis and EMT

**Fig. 6:**
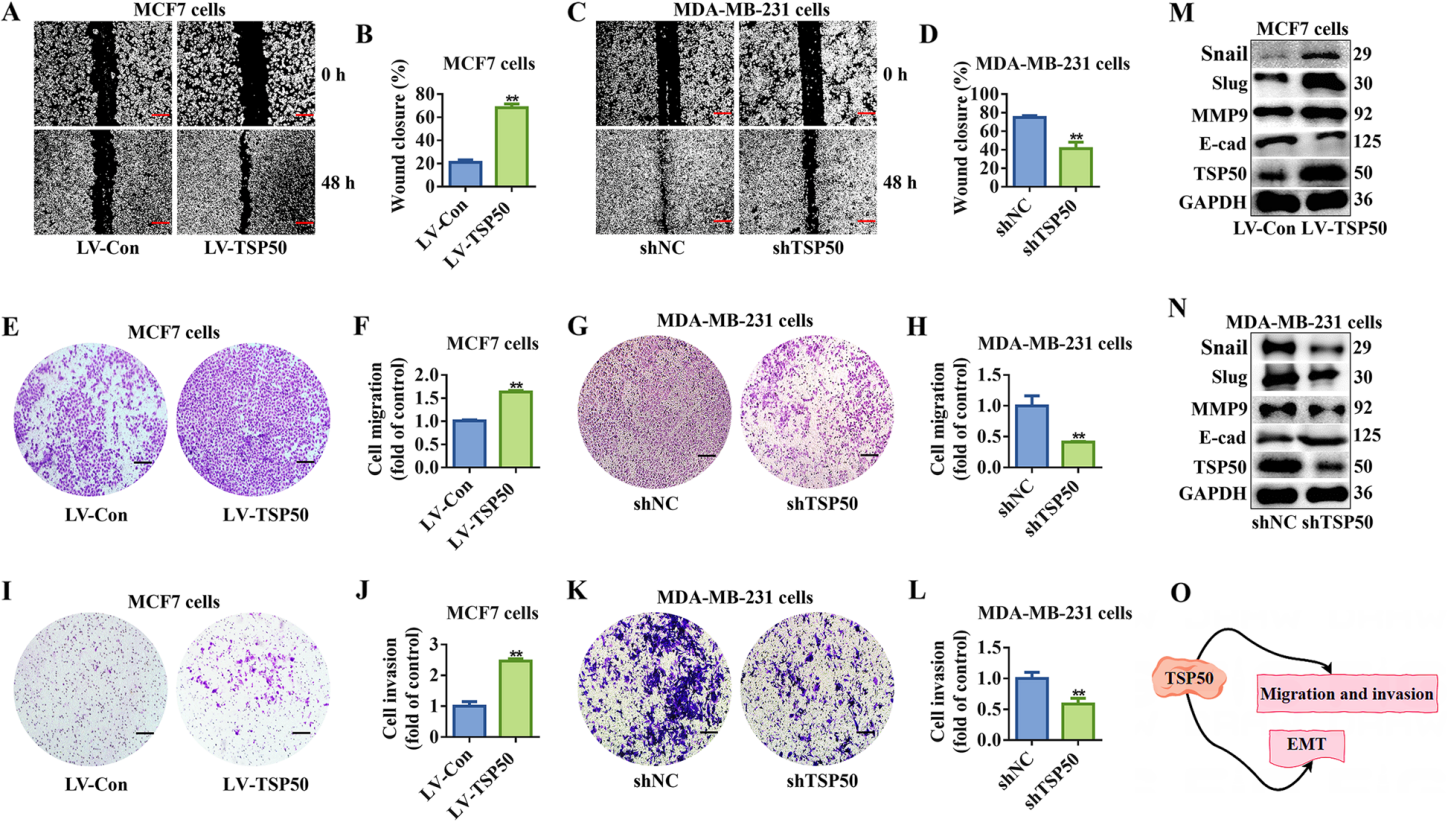
TSP50 promotes breast cancer cell metastasis and EMT. **A**, **B** Wound healing of MCF7 cells stably overexpressing NC or TSP50. **C**, **D** Wound healing of MDA-MB-231 cells transfected with shNC or shTSP50. **E**, **F** Migration of MCF7 cells stably overexpressing NC or TSP50. **G**, **H** Migration of MDA-MB-231 cells transfected with shNC or shTSP50. **I**, **J** Invasion of MCF7 cells stably overexpressing NC or TSP50. **K**, **L** Invasion of MDA-MB-231 cells transfected with shNC or shTSP50. **M** The protein expression of EMT-related markers E-cad, MMP9, Slug and Snail in MCF7 cells stably overexpressing NC or TSP50. **N** The protein expression of EMT-related markers E-cad, MMP9, Slug and Snail in MDA-MB-231 cells transfected with shNC or shTSP50. **O** Schematic diagram of TSP50, which promotes the migration, invasion and EMT of breast cancer cells. Scale bar, 25 μm. *N* = 3 biologically independent replicates. Student’s t-test statistical analysis was used (**p < *0.01*)

Metastasis is the primary cause of mortality in breast cancer patients. Therefore, we investigated TSP50’s role in breast cancer metastasis. According to the results of investigations on wound healing, breast cancer cells that overexpressed TSP50 repaired wounds more quickly than the control cells (Fig. 6A and B, Fig. S4A and B). However, TSP50 knockdown breast cancer cells showed the opposite behavior (Fig. 6C and D, Fig. S4C and D). Studies on migration and invasion revealed that, in contrast to TSP50 knockdown, TSP50 overexpression significantly boosted both cell migration and invasion (as measured by the Transwell assay and Matrigel experiment, respectively) (Fig. 6E-L, Fig. S4E-L). Moreover, studies have revealed that EMT cells have CSC-like properties, and CSCs exhibit a mesenchymal-like phenotype [38]. We further investigated the role of TSP50 in EMT regulation of breast cancer cells. Western blot analysis was used to examine the expression of EMT-related markers, and the results showed that when TSP50 was overexpressed in breast cancer cells, E-cadherin protein level was dramatically reduced, whereas MMP9, Slug and Snail protein levels were significantly elevated (Fig. 6M, Fig. S4M). In contrast to control cells, TSP50 knockdown breast cancer cells showed significantly increased E-cadherin protein level and significantly decreased MMP9, Slug and Snail protein levels (Fig. 6N, Fig. S4N). However, variations in mRNA levels of the EMT-related markers were inconsistent (Fig. S1K and L, Fig. S4O and P). Furthermore, we evaluated the expression levels of EMT-related markers in tumor tissues formed by limited dilution and tumorigenesis assay using breast cancer cells with TSP50 stably overexpressed or knocked down. As shown in Fig. S1M-T, the alterations in the expression levels of EMT-related markers in tumor tissues formed by TSP50 overexpression or knockdown of breast cancer cells matched our cell-based detection findings. These results demonstrate that TSP50 promoted the metastasis and EMT of breast cancer cells (Fig. 6O).

### TSP50 and PI3K p85α regulatory subunit competitively bind with PI3K p110α catalytic subunit to enhance its catalytic activity

**Fig. 7:**
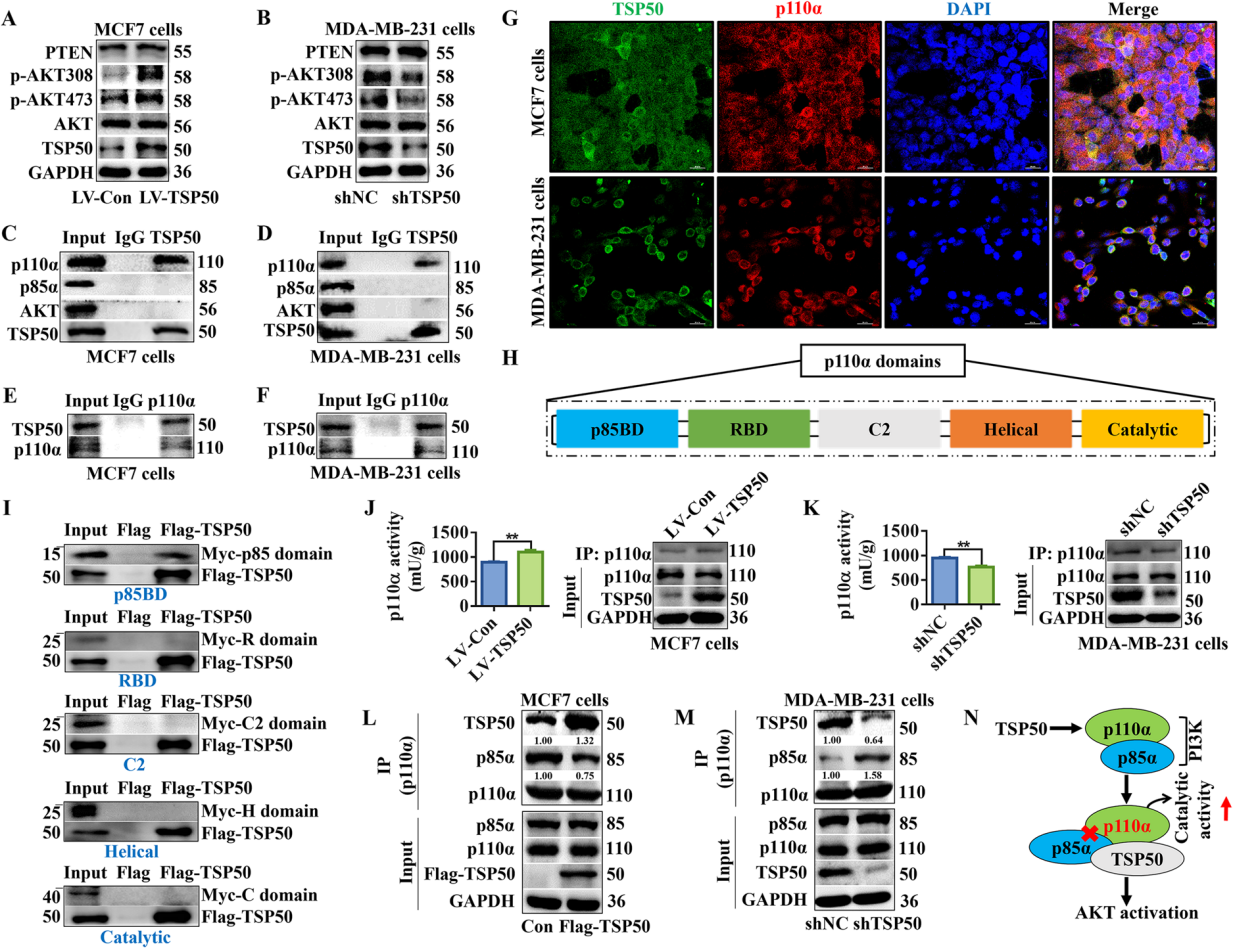
TSP50 and PI3K p85α regulatory subunit competitively bind with PI3K p110α catalytic subunit to enhance its catalytic activity. **A**, **B** Western blot detection results of PI3K/AKT signal-related markers expression in MCF7 cells stably overexpressing NC or TSP50 and MDA-MB-231 cells transfected with shNC or shTSP50. **C**, **D** MCF7 and MDA-MB-231 cells were harvested and subjected to Co-IP with anti-TSP50 antibody, followed by Western blot analysis with anti-AKT, anti-p85α or anti-p110α antibody. **E**, **F** MCF7 and MDA-MB-231 cells were harvested and subjected to Co-IP with anti- p110α antibody, followed by Western blot analysis with an anti-TSP50 antibody. **G** MCF7 and MDA-MB-231 cells were fixed and subjected to IF analysis. TSP50 co-localizes with p110α in MCF7 and MDA-MB-231 cells. Scale bar, 50 μm. **H** The five domains in the p110α subunit. **I** In vitro Co-IP analysis of different domains of p110α (p85BD, RBD, C2, helical, and catalytic domains) with Flag-TSP50. The p85BD domain of p110α interacts with TSP50. **J**, **K** The p110α was purified from MCF7 cells stably overexpressing NC or TSP50 and MDA-MB-231 cells transfected with shNC or shTSP50. The catalytic activity of p110α was measured. **L**, **M** MCF7 cells stably overexpressing NC or TSP50 and MDA-MB-231 cells transfected with shNC or shTSP50 were harvested and subjected to Co-IP with anti-p110α antibody, followed by Western blot analysis with anti-p85α and anti-TSP50 antibodies. **N** A model of TSP50 regulating PI3K/AKT signaling through interaction with p110α. *N* = 3 biologically independent replicates. Student’s t-test statistical analysis was used. **p < *0.01*

The EMT of breast cancer cells is regulated by a variety of signaling pathways, including Wnt/β-catenin [39], Notch [40], Hedgehog [41] and PI3K/AKT [42], which have been confirmed to be involved in CSC-like properties maintenance. Therefore, we examined the effects of TSP50 on the activation of these pathways. The IF assay results showed that overexpression or knockdown of TSP50 did not affect the nuclear translocation of β-catenin (Wnt/β-catenin signal factor) or NCID (Notch signal factor) in breast cancer cells (Fig. S5A-D). Additionally, the expression levels of SHH and PTCH1, two essential components of the Hedgehog pathway, were also not regulated by TSP50 (Fig. S5E and F). These results indicate that TSP50 does not mediate the activation of the Wnt/β-catenin, Notch or Hedgehog pathways. However, we found significantly altered p-AKT levels in response to the overexpression or suppression of TSP50 in breast cancer cells, with constant AKT levels, indicating that PI3K/AKT signaling is stimulated by TSP50 (Fig. 7A and B, Fig. S5G and H).

We further investigated how TSP50 activated PI3K/AKT signaling. Studies have shown that PTEN is an important regulatory factor in PI3K/AKT signal [43]. In this study, Western blot analysis results showed that PTEN expression remained stable after TSP50 was overexpressed or knocked down in breast cancer cells (Fig. 7A and B, Fig. S5G and H). PI3Ks are a family of lipid kinases (Class I, II and III), among which class I PI3K is the most widely studied. Class I PI3K can be formed by different catalytic subunits, namely p110α, p110β, p110γ or p110δ, which are involved in the production of phosphatidylinositol 3-phosphate (PI3P) [44]. The most characterized catalytic and regulatory subunits of PI3K, p110α and p85α, are closely associated with cancer progression and tumorigenesis [45]. The p110α kinase, which is involved in the production of PI3P from phosphatidylinositol 4,5-biphosphate (PI2P), activates the AKT signaling cascade [46]. We speculated that TSP50 might bind with the aforementioned PI3K/AKT key factors, such as p85α, p110α and AKT, which were selected for Co-IP analysis to identify potential target molecules regulated by TSP50. The results showed that endogenous TSP50 interacted with p110α rather than with p85α or AKT (Fig. 7C-F, Fig. S5 I and J). As confirmed by IF, TSP50 and p110α were colocalized in the cytoplasm of MCF7 and MDA-MB-231 cells (Fig. 7G). The p110α subunit consists of p85 binding domain (p85BD), Ras-binding domain (RBD), C2, Helical and Catalytic domains (Fig. 7H). The MyC-tagged domain plasmids were synthesized and co-transfected with Flag-TSP50 overexpression plasmid into MDA-MB-231 cells for Co-IP analysis. Our results showed that the p85BD was responsible for the interaction of p110α with TSP50 (Fig. 7I). Next, the p110α protein was purified from breast cancer cells with TSP50 overexpressed or knocked down using an IP assay, and its activity was then determined. We found that compared with the control group, p110α activity was significantly increased when TSP50 was overexpressed, while decreased markedly in TSP50 knockdown breast cancer cells (Fig. 7J and K). It is confirmed that the increased disruption of p110α binding to the inhibitory subunit p85α relieves its catalytic inhibition, and increases p110α membrane lipid binding [47]. Intriguingly, we have demonstrated that TSP50 binds to p110α through p85BD, however, p85α was not detected in the complex obtained using Co-IP with anti-TSP50 (Fig. 7C, D and I). On the basis of the findings, we hypothesized that TSP50 might enhance PI3K activity by reducing complex formation between p85α subunit and p110a. To verify this hypothesis, we analyzed the effect of TSP50 on the p110α-p85α interaction. When TSP50 was overexpressed, the interaction between p110a and p85α was reduced, and the level of TSP50 bound to p110a increased significantly in the complex obtained by Co-IP assay. Meanwhile, an enhanced p110α-p85α interaction was found in TSP50 knockdown cells compared with the shNC group, accompanied with a reduced TSP50 binding level to p110α (Fig. 7L and M, Fig. S5 K and L). Therefore, these data indicate that TSP50 positively modulates PI3K activity by preventing p85α from binding to p110a (Fig. 7N).

### The TSP50 D206A mutation enhances the binding ability of p85α with p110α, thereby inhibiting the activity of p110α

**Fig. 8:**
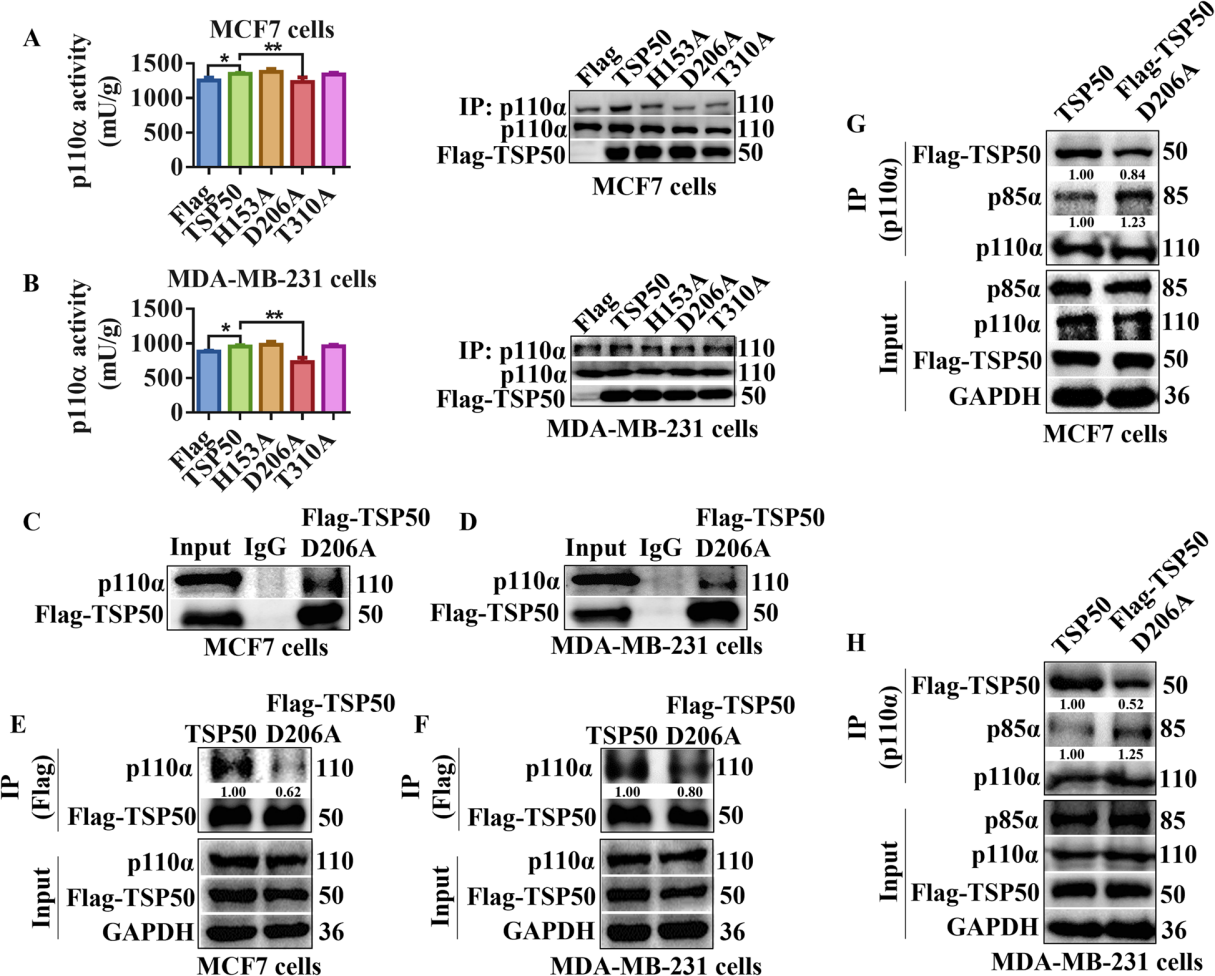
TSP50 D206A mutation enhances the binding ability of p85α with p110α. **A**, **B** The p110α was purified from MCF7 and MDA-MB-231 cells transfected with wild-type TSP50, H153A, D206A or T310A mutant. The catalytic activity of p110α was measured. **C**, **D** MCF7 and MDA-MB-231 cells transfected with Flag-TSP50 D206A mutant were harvested and subjected to Co-IP with anti-Flag antibody, followed by Western blot analysis with anti-p110α antibody. **E**, **F** MCF7 and MDA-MB-231 cells transfected with Flag-TSP50 wild-type or Flag-TSP50 D206A mutant were harvested and subjected to Co-IP with an anti-Flag antibody, followed by Western blot analysis with the anti-p110α antibody. **G**, **H** MCF7 cells and MDA-MB-231 cells transfected with Flag-TSP50 wild type or Flag-TSP50 D206A mutant were harvested and subjected to Co-IP with anti-p110α antibody, followed by Western blot analysis with anti-p85α and anti-Flag antibodies. *N* = 3 biologically independent replicates. Student’s t-test or one-way ANOVA statistical analysis was used. ^*^p < *0.05* and **p < *0.01*

Our previous study has shown that the catalytic triad of TSP50 is essential for its function in cell proliferation, and mutants (H153A, D206A or T310A) in the catalytic triad abolish the enzyme activity of TSP50 [48]. Therefore, we investigated the effects of different TSP50 mutants on p110α activity and found that p110α activity was significantly decreased only in TSP50 D206A mutated breast cancer cells compared with the TSP50 wild-type group (Fig. 8A and B). Further analysis results revealed that TSP50-p110α binding was weakened by the D206A mutation in TSP50 (Fig. 8C-F). These results indicate that the TSP50 D206A mutation may affect its binding ability with p110α through a conformational change rather than an activity alteration in TSP50. We also analyzed the effect of TSP50 D206A mutant on the p110α-p85α interaction, and the results showed that p110α and p85α interacted more favorably in breast cancer cells with the TSP50 D206A mutation compared with the TSP50 wild-type breast cancer cells (Fig. 8G and H), further verifying the mechanism by which TSP50 activates PI3K/AKT signal.

### TSP50-mediated PI3K/AKT signaling activation is essential for maintaining CSC-like properties, EMT and metastasis in breast cancer cells

**Fig. 9:**
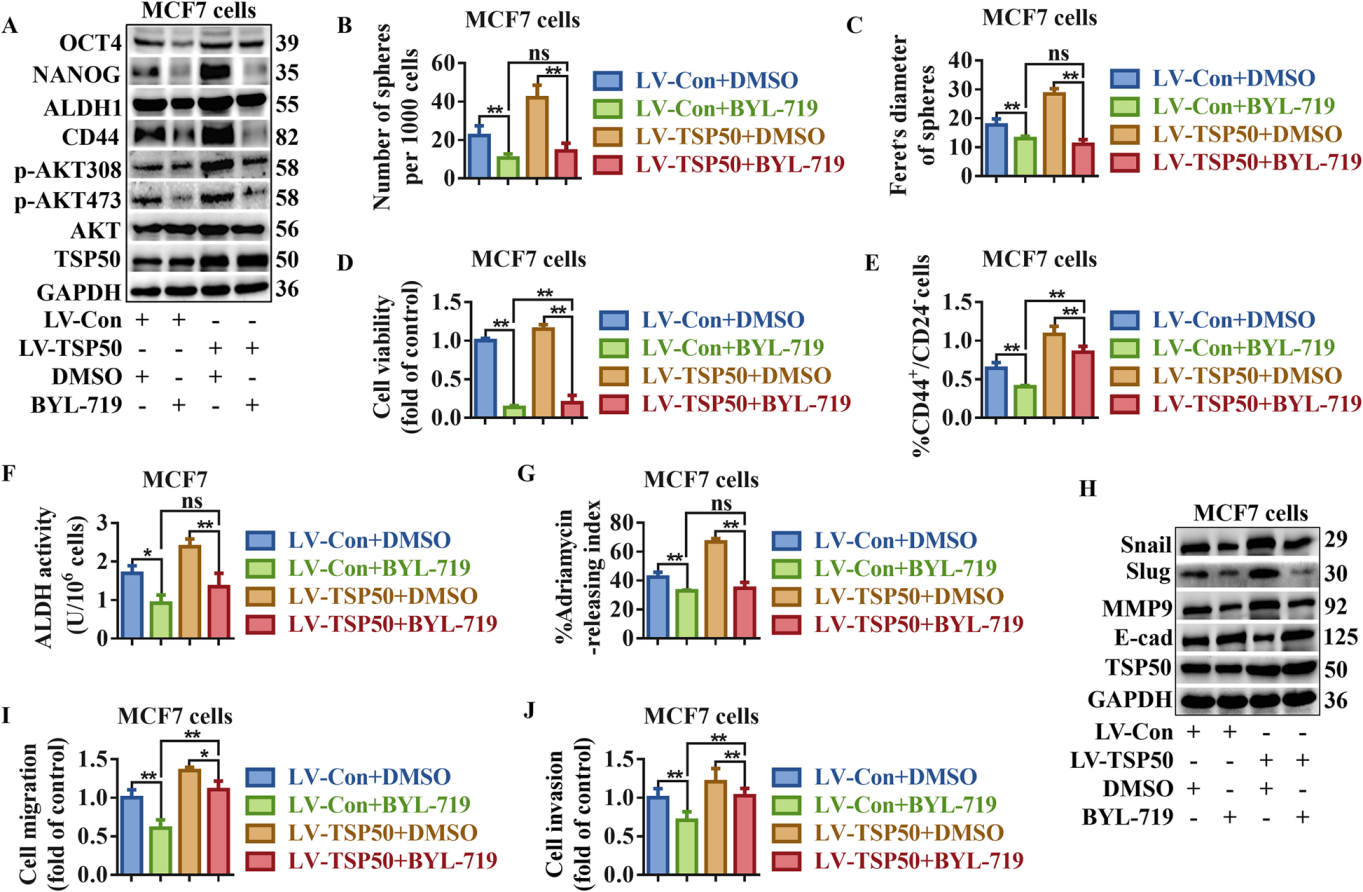
Inhibition of PI3K/AKT signaling partially reversed TSP50-mediated CSC-like properties, EMT and metastasis maintenance in MCF7 cells. TSP50 or NC stably overexpressed MCF7 cells were treated with BYL-719. **A** The levels of indicated BCSC-related markers were determined by Western blot. **B**, **C** Primary mammosphere number and size were calculated. **D** The number of colonies analysis results. **E** The subpopulation proportion analysis results of CD44^+^/CD24^−^ cells. **F** ALDH activity detection results. **G** The ADR pumping rate. **H** The levels of EMT-related markers were determined by Western blot. **I** Cell migration detection results. **J** Cell invasion detection results. *N* = 3 biologically independent replicates. Student’s t-test or one-way ANOVA statistical analysis was used. ^*^p < *0.05*, **p < *0.01* and ns, no significance

**Fig. 10:**
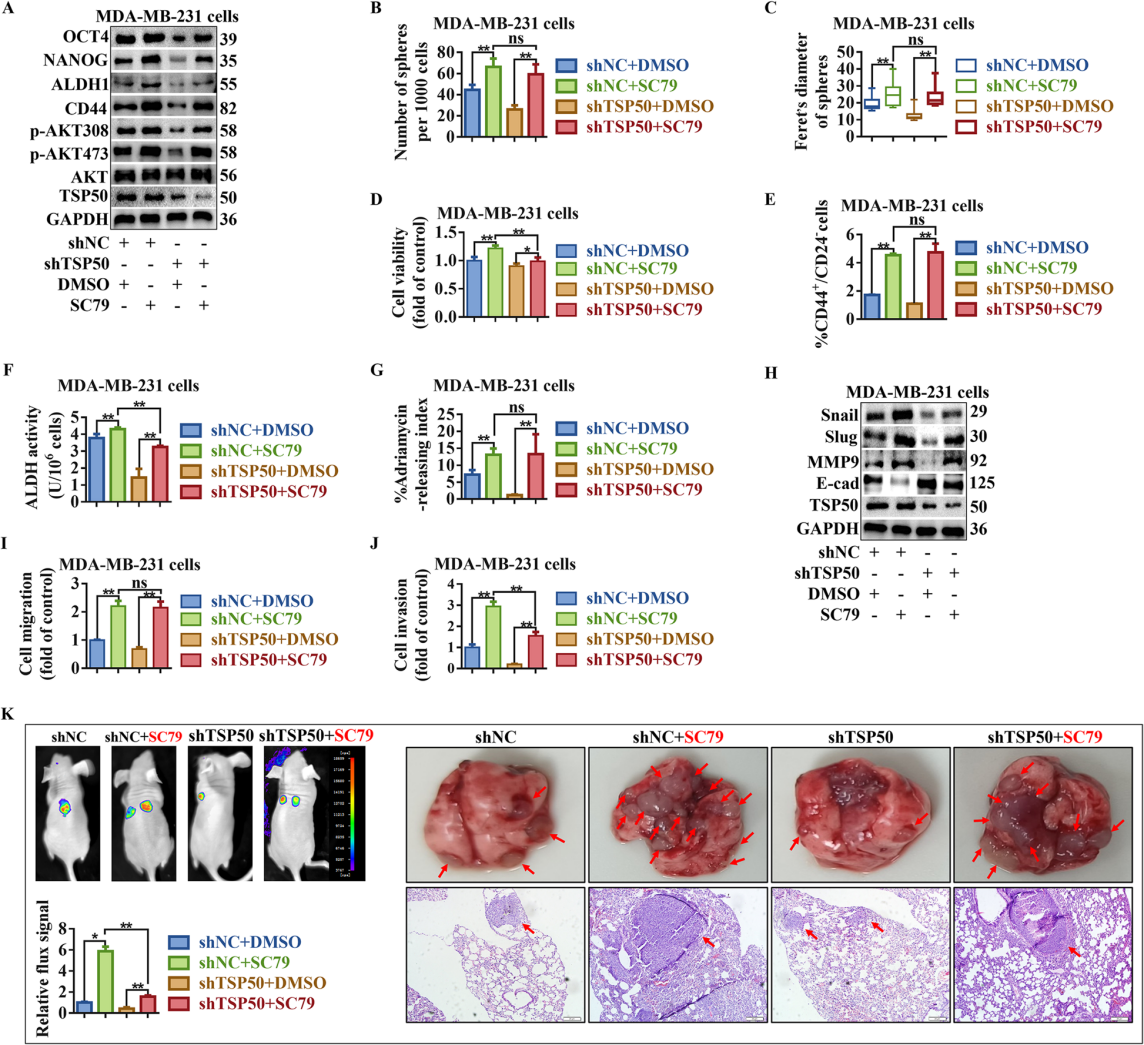
PI3K/AKT signaling activation partially restored the CSC-like properties, EMT and metastasis inhibited by TSP50 knockdown. The shNC or shTSP50 transfected MDA-MB-231 cells were treated with SC79. **A** The levels of indicated BCSC-related markers were determined by Western blot. **B**, **C** Primary mammospheres number and size were calculated. **D** The number of colonies analysis results. **E** The subpopulation proportion analysis results of CD44^+^/CD24^−^ cells. **F** ALDH activity detection results. **G** The ADR pumping rate. **H** The levels of EMT-related markers were determined by Western blot. **I** Migration assay detection results. **J** Cell invasion detection results. **K** Bioluminescence images of lung metastasis in mice that were injected with cells through the tail veins as indicated, and the metastasis was quantified by measuring the photo flux. Macroscopically or HE staining was used to examine lung metastatic nodules. The red arrows denoted the metastatic nodules. *N* = 3 biologically independent replicates. Student’s t-test or one-way ANOVA statistical analysis was used. ^*^p < *0.05*, **p < *0.01* and ns, no significance

We treated cells with the p110α inhibitor BYL-719, AKT inhibitor LY294002, or AKT agonist SC79 to determine whether TSP50 promotes breast cancer CSC-like traits, EMT and metastasis through the PI3K/AKT signaling pathway. As shown in Fig. 9A, S6A, S7A and B, BYL-719 or LY294002 treatment inhibited the phosphorylation level of AKT and partly reversed the TSP50-induced promotion of AKT phosphorylation and CSC-like marker expression. BYL-719 or LY294002 treated cells exhibited a decrease in sphere formation efficiency (both number and diameter), the number of colonies, the proportion of CD44^+^/CD24^−^ cells, ALDH activity and ADR pumping rate, even in the presence of TSP50 (Fig. 9B-G, Fig. S8A and B, Fig. S6B-G, Fig. S8C and D, Fig. S7C-G, Fig. S8E and F). Treatment with BYL-719 or LY294002 also reversed the decreased expression of E-cadherin and increased expression of MMP9, Slug and Snail induced by TSP50 (Fig. 9H, Fig. S6H and Fig. S7H and I), and impaired TSP50-induced cell migration and invasion (Fig. 9I and J, Fig. S9A and B, Fig. S6I and J, Fig. S9C and D, Fig. S7J and K, Fig. S9E and F). In addition, SC79 enhanced the phosphorylation level of AKT, meanwhile, SC79 treatment restored AKT phosphorylation and CSC-like marker protein levels (Fig. 10A, Fig. S10A), CSC-like properties (Fig. 10B-G, Fig. S11A and B, Fig. S10B-G, Fig. S11C and D), EMT level (Fig. 10H, Fig. S10H), cell migration and invasion activity (Fig. 10I and J, Fig. S12A and B, Fig. S10I and J, Fig. S12C and D) and tumor metastasis in vivo (Fig. 10K) after TSP50 knockdown in breast cancer cells. These results reveal that TSP50 increases breast cancer CSC-like traits, EMT and metastasis partially through the PI3K/AKT signaling pathway.

### PI3K/AKT signaling activation is crucial for TSP50 tumor-promoting effects in vivo

**Fig. 11:**
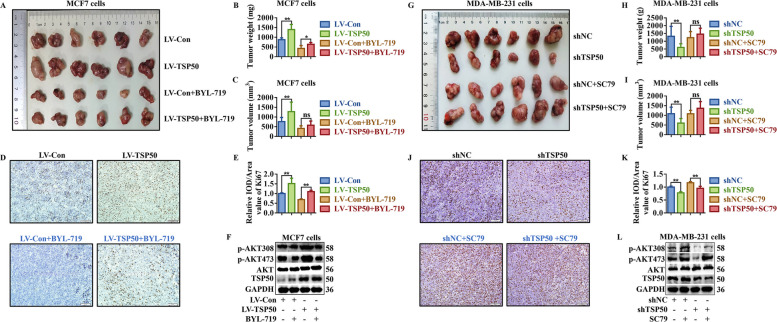
TSP50 promotes tumorigenesis through PI3K/AKT signaling in vivo. **A-F** The nude mice were injected with NC or TSP50 stably overexpressed mammospheres and then treated with BYL-719. Photography of xenograft tumor (**A**), tumor weight (**B**), tumor volume (**C**), Ki67 level (**D**, **E**) and PI3K/AKT signal-related markers levels (**F**). **G-L** The nude mice were injected with stable shNC or TSP50-knockdown mammospheres and then treated with SC79. Photography of xenograft tumor (**G**), tumor weight (**H**), tumor volume (**I**), Ki67 level (**J**, **K**) and PI3K/AKT signal-related markers levels (**L**). Scale bar, 50 μm. *N* = 3 biologically independent replicates. Student’s t-test statistical analysis was used. ^*^p < *0.05*, **p < *0.01* and ns, no significance

To explore the effect of TSP50 on pathological progression of breast cancer, an in vivo tumor formation assay was performed (*n* = 6/group). The tumor weight, volume, Ki67 and p-AKT levels were higher in TSP50 stable overexpression group than in the control group (Fig. 11A-F, Fig. S13A-F). As expected, BYL-719 or LY290042 treatment reversed the tumorigenic effects of TSP50 and the TSP50-induced promotion of AKT phosphorylation in tumor tissues (Fig. 11A-F, Fig. S13A-F). In contrast, in MDA-MB-231-derived mammospheres, TSP50 knockdown significantly inhibited tumor formation, accompanied with a dramatic reduction in Ki67 and p-AKT levels (Fig. 11G-L). Xenografted mice treated with SC79 reversed the tumorigenesis inhibition mediated by TSP50-knockdown through enhancing the phosphorylation level of AKT (Fig. 11G-L).

### TSP50 expression is positively correlated with p-AKT and ALDH1 levels in human breast tumor tissues

**Fig. 12:**
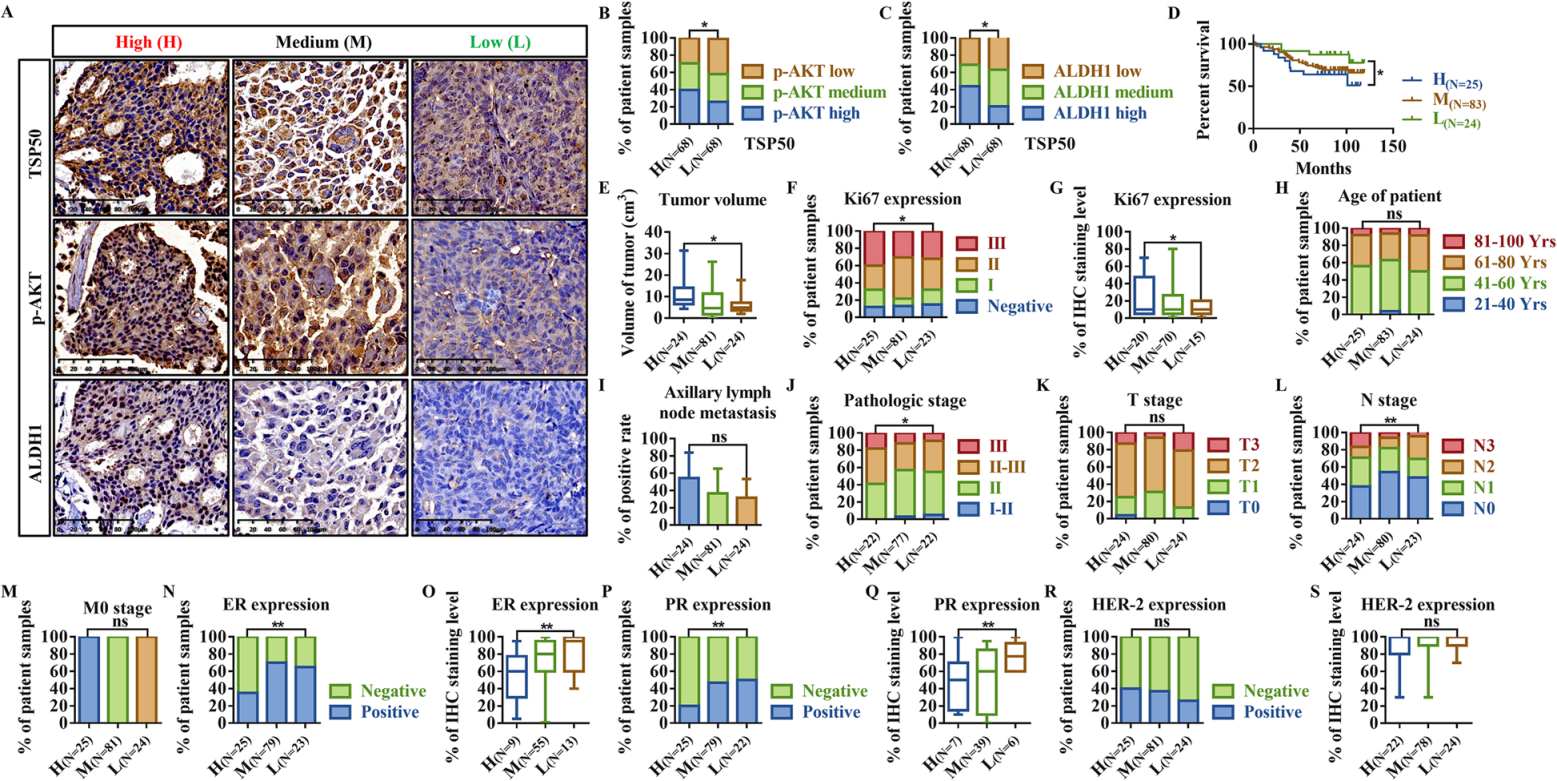
TSP50 expression is positively correlated with p-AKT and ALDH1 levels, and the TSP50/p-AKT/ALDH1 positive relationship is a potential diagnostic index for human breast cancer. **A** TSP50, p-AKT and ALDH1 expression levels were analyzed using IHC in human breast cancer tissues (*n* = 136). Scale bar, 100 μm. **B**, **C** The expression levels of p-AKT and ALDH1 were positively correlated with TSP50 expression levels. Cancer tissues with high TSP50 expression (*n* = 68) and low TSP50 expression (*n* = 68) were analyzed using IHC. **D-S** The relationship analysis results between TSP50/p-AKT/ALDH1 expression and patient survival time (**D**), tumor volume (**E**), Ki67 level (**F**, **G**), age (**H**), axillary lymph node metastasis (**I**), pathologic stage (**J**), TNM stage (**K-M**), ER level (**N**, **O**), PR level (**P**, **Q**) and HER-2 level (**R**, **S**) in human breast cancer tissues. Spearman, Chi-square or one-way ANOVA statistical analysis was used. ^*^p < *0.05*, **p < *0.01* and ns, no significance

Based on the finding that TSP50 regulates PI3K/AKT signaling and promotes CSC-like phenotypes in breast cancer cells, we investigated whether TSP50 is correlated with the levels of p-AKT and ALDH1 in breast cancer specimens. The expression levels of TSP50, p-AKT and ALDH1 were examined in 136 breast cancer tissues using IHC (Fig. 12A). As expected, p-AKT and ALDH1 protein levels in 136 breast cancer specimens increased significantly with increasing TSP50 expression (p-AKT and TSP50, *r* = 0.42, *p* < 0.01; ALDH1 and TSP50, *r* = 0.46, *p* < 0.01). In particular, a significant proportion of the samples with high TSP50 expression also exhibited elevated levels of p-AKT (40%, 27/68) and ALDH1 (44%, 30/68) (Fig. 12B and C). Accordingly, lower levels of p-AKT and ALDH1 were found in 41% (28/68) and 37% (25/68) of the samples with low TSP50 expression (Fig. 12B and C). Collectively, these findings provide compelling evidence that TSP50 expression positively correlates with p-AKT and ALDH1 protein levels in human breast cancer.

### The positive relationship between TSP50, p-AKT and ALDH1 is a potential diagnostic index for human breast cancer

The clinical significance of the positive correlations between TSP50, p-AKT, and ALDH1 in human breast cancer were investigated. We first analyzed whether TSP50, p-AKT and ALDH1 had a synergistic effect on patient survival, tumor size, Ki67 levels and axillary lymph node metastasis. According to the statistics, patients exhibiting low expression levels of TSP50/p-AKT/ALDH1 demonstrated significantly higher survival rates. (Fig. 12D). Furthermore, our observations indicated that tumor volumes in patients with high TSP50/p-AKT/ALDH1 expression were 2-times larger than those in patients with low TSP50/p-AKT/ALDH1 expression, which was consistent with the comparable changes in Ki67 levels (Grade III, 40% (10/25) vs. 30% (7/23)) (Fig. 12E-G). However, our analysis did not reveal any significant association between the expression levels of TSP50/p-AKT/ALDH1 and clinical parameters such as patient age or the presence of axillary lymph node metastases (Fig. 12H and I). Moreover, the TSP50/p-AKT/ALDH1 expression levels were strongly correlated with tumor pathological stage (pathological stage III, TSP50/p-AKT/ALDH1 high group 18% (4/22) vs. TSP50/p-AKT/ALDH1 low group 9% (2/22)) and N stage (N2-N3 stages, TSP50/p-AKT/ALDH1 high group 17% (4/24) vs. TSP50/p-AKT/ALDH1 low group 4% (1/23)), with the exception of T and M stages (Fig. 12J-M). We then investigated whether the expression of clinical prognosis and therapy molecular markers, such as ER, PR, and HER-2 [49], were associated with TSP50/p-AKT/ALDH1 levels. As illustrated in Fig. 12N-S, 65% (15/23) of breast cancer patients expressing low TSP50/p-AKT/ALDH1 showed ER positivity, and 50% (11/22) of breast cancer patients expressing low TSP50/p-AKT/ALDH1 also showed PR positivity, with a higher IHC staining level. And for HER-2, no significant discernible differences were detected. Based on the above clinical data, TSP50/p-AKT/ALDH1 levels can be used as a possible diagnostic index for early breast cancer.

## Discussion

Breast cancer, which is the second leading cause of cancer-related mortality, maintains to be a significant burden for women globally [50]. Breast cancer patients frequently experience metastasis, treatment resistance and tumor recurrence, which are proposed to be regulated by a small population of CSCs, thus causing a generally poor prognosis [51–53]. Studies have shown that breast cancer treatment efficiency can be improved by targeting CSCs in combination with conventional chemotherapy [54, 55]. Notably, spontaneous acquisition of CSC-like features in cancer cells is usually accompanied with EMT. Therefore, understanding the regulatory mechanisms of CSCs and EMT is of great significance for identifying potential targets for CSC-specific therapies. Our previous studies identified TSP50 as a key driver of breast cancer cell proliferation and invasion, which is responsible for aggressive behavior and poor prognosis during breast cancer progression [13, 14]. Here, we report a previously unidentified role of TSP50 in CSC-like phenotypes and EMT regulation in breast cancer cells. The results demonstrate that the oncogene TSP50 plays an important role in CSC-like phenotypes and EMT in breast cancer cells. Additionally, we reveal an underlying molecular mechanism that TSP50 activates the PI3K/AKT signaling pathway, leading to the promotion of CSC-like phenotypes and EMT in breast cancer cells.

The level of TSP50 holds significant clinical implications for individuals with breast cancer [20]. According to our analysis, TSP50 was expressed differently in distinct breast cancer cells and molecular subtypes, and highly expressed TSP50 was significantly associated with the shorter OS and DMFS, indicating that TSP50 may function as a potential diagnostic indicator for breast cancer and deserves further experimental validation. Interestingly, the ROC plotter analysis showed that non-responders to chemotherapy harbored higher TSP50 expression, thus, TSP50 can be considered as a biomarker of resistance to chemotherapy in breast cancer. However, the AUC value of the post-chemotherapy 5-year PFS prediction just reached 0.585.

The CD44^+^/CD24^−^ cells have been identified as BCSCs [56]. Particularly, numerous lines of evidence suggest that elevated CD44 expression is associated with the CSC-like phenotypes across various tumor types [57–59]. Furthermore, ALDH1, NANOG and OCT4 have been confirmed to be the key BCSC markers. ALDH1-highly-expressed breast cells are indicative of possessing stem or progenitor properties with wide-ranging differentiation potential and superior growth performance [60]. The transcription factor OCT4, along with Nanog, can serve as markers for embryonic stem cell (ESC), which are crucial for sustaining the pluripotent self-renewal of ESCs [61]. Under hypoxic conditions, increased ALDH transcription and elevated OCT4 and Nanog expression facilitate the reprogramming of non-stem cancer cells to a CSC-like phenotype [62, 63]. In this study, we found that TSP50 significantly increased the CD44^+^/CD24^−^ subpopulation of breast cancer cells. Further bioinformatics analysis of breast cancer based on TCGA database and the in vitro and in vivo detection results showed that TSP50 can regulate the expression of key BCSC factors, such as CD44, ALDH1, NANOG and OCT4. By upregulating the expression of these BCSC factors, TSP50 promoted the formation capacity of primary and secondary mammospheres, increased the level of colony formation and ADR drug resistance, and enhanced the tumorigenicity of breast cancer cells. Our findings suggest a potential role of TSP50 in regulating CSCs in breast cancer cells. The single cell analysis enables a more nuanced investigation at the individual cell level, potentially revealing heterogeneity within the cell population. We believe that it is of great significance for further investigation on the expression of TSP50 and the development of CSC phenotype, and will be conducted in further study in the future.

It has been indicated that EMT is associated with the development of stemness traits [64, 65]. Therefore, the effect of TSP50 on EMT in breast cancer cells was analyzed. Both E-cadherin [66–68], a key epithelial cell marker and MMP9 [69, 70], one of most widely studied matrix metalloproteinases (MMPs), have been proposed to induce EMT and play a crucial role in the development of malignant tumors. By suppressing E-cadherin and activating MMPs, the Snail family (Snail and Slug) are served as the primary regulator of EMT [71]. Our studies showed that TSP50 promoted the expression of Slug and Snail, leading to the downregulation of E-cadherin and elevated expression of MMP9 both in vivo and in vitro. Additionally, the results of the wound healing, migration and invasion assays showed that TSP50 is involved in the regulation of the migration and invasion abilities of breast cancer cells. These findings indicate that EMT is triggered by TSP50 and may subsequently induce CSC-like phenotypes in breast cancer cells.

Currently, the cellular mechanisms underlying the maintenance of CSCs regulation are poorly understood, despite accumulating evidence that the PI3K/Akt signaling pathway is essential for prostate CSCs maintenance [72]. In breast cancer, PI3K/Akt activation by PTEN knockdown is known to enrich BCSCs [73]. In contrast, the CSC-like populations are decreased by inhibiting the PI3K pathway using certain inhibitors in prostate cancer cells [72]. These studies clearly suggest that the PI3K/AKT pathway is indispensable for the maintenance of CSCs and targeting PI3K signaling may be prudent in cancer therapy [74]. To determine whether TSP50 functions through the PI3K/AKT signaling pathway, we treated TSP50-overexpressed breast cancer cells with the PI3K/AKT signaling inhibitor LY294002 and TSP50-knockdowned breast cancer cells with the agonist SC79 for rescue experiments. The results showed that TSP50 functions through the PI3K/AKT signaling pathway to regulate the expression of BCSC and EMT markers. Mammosphere formation, ADR resistance, migration, invasion and tumorigenic assays results further confirmed that TSP50 acts through the PI3K/AKT signaling pathway. Mechanistically, TSP50 was shown to interact with the p110α subunit, potentially enhancing its catalytic activity. However, p85α was not found in the precipitated complex in the Co-IP assay with anti-TSP50. Interestingly, p110α p85 binding domain was responsible for the interaction of p110α with TSP50. And overexpression of TSP50 reduced the binding of p110α with p85α, conversely, TSP50 knockdown increased the binding level of p110α with p85α. The most well-studied PI3K catalytic and regulatory subunits, p110α and p85α, are closely associated with cancer progression and tumorigenesis [45]. Studies have shown that the p85 subunit attaches to the p110 subunit and suppresses its catalytic activity at rest or in the absence of stimulation [75]. The p110/p85 complex is recruited to the plasma membrane in response to stimulation by, such as, growth factors, where p85 binds via its SH2 domains to tyrosine-phosphorylated receptor tyrosine kinases (RTKs) or adaptor proteins, dislodging p85 from the PI3K complex and activating p110 [76]. Hence, we suggest that the competitive binding of TSP50 and p85α with p110α results in the elevation of p110α activity, which ultimately activates PI3K/AKT signaling. This was further confirmed by the addition of BYL-719, the p110α inhibitor, which reversed the CSC-like phenotypes maintenance and EMT promotion effects of TSP50 in breast cancer cells.

The enzymatic activity of TSP50 is reduced by the H153A, D206A or T310A mutation [48]. In the present study, we analyzed the effect of the altered TSP50 on the activity of p110α and found that p110α activity was solely inhibited by the TSP50 D206A mutant. In addition, compared with the TSP50 wild-type group, TSP50 D206A mutant overexpressed breast cancer cells showed increased p110α binding ability with p85α. These findings imply that the TSP50 D206A mutation may inhibit the activity of p110α by altering the conformation of TSP50 rather than its activity. Furthermore, TSP50 may regulate CSCs through a variety of pathways, although we demonstrated that TSP50 has no significant effect on classical CSC-related Wnt/β-catenin, Notch and Hedgehog signaling. Are these pathways independent or overlapping? These issues require further investigation.

The clinical value of Akt activation and ALDH1 abnormal expression has been widely reported in human breast cancer [77–80]. In the present study, TSP50 expression and p-AKT or ALDH1 levels in breast cancer specimens were positively correlated. Previous studies have indicated that ER- and PR-positive patients appear to have a better prognosis in terms of both survival rates and response to hormone treatment, while HER-2 was identified as a poor prognostic factor [81, 82]. Here, we demonstrated that patients exhibiting low levels of TSP50/p-AKT/ALDH1 were more likely to have ER- and PR-positive tumors. Nevertheless, there was no significant difference in the expression of HER-2. Therefore, our results provide a new reference for the prognosis and treatment of patients with breast cancer.

## Conclusion

**Fig. 13:**
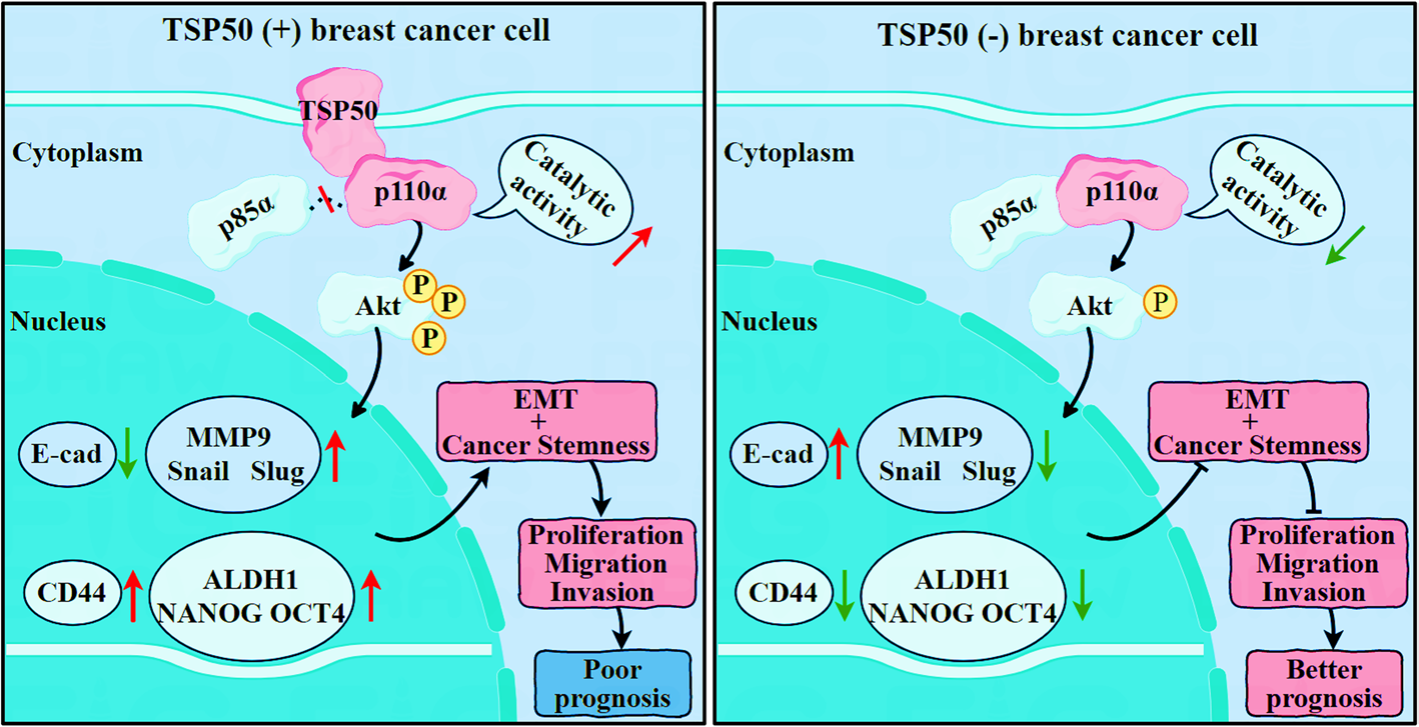
Schematic illustration of the role and molecular mechanisms of TSP50 in CSC-like properties and EMT in breast cancer. In breast cancer cells, TSP50 and p85α can competitively bind with p110α, thereby increasing the activity of p110α and resulting in an increase in AKT phosphorylation levels, which then mediate PI3K/AKT signal transduction. TSP50 promotes the expression of CSCs and EMT markers through the above-mentioned regulatory mechanisms and enhances the CSC-like properties and EMT of breast cancer cells, which are beneficial for the proliferation, migration and invasion of tumor cells. Furthermore, TSP50 is positively correlated with PI3K/AKT signaling and CSC-like properties in human breast cancer tissues, leading to the poor clinical prognosis

In summary, TSP50 and p85α competitively bind with p110α, which increases the catalytic activity of p110α to enhance PI3K/AKT signal transduction, thereby promoting CSC-like properties and EMT, which is beneficial for the migration and invasion of breast cancer cells. Furthermore, the positive correlation between TSP50 and p-AKT/ALDH1 is associated with clinicopathological features (Fig. 13). We reveal a novel regulatory mechanism for TSP50 in breast cancer progression. These results may provide new ideas for TSP50-targeted breast cancer therapy.

## Supplementary Information

Supplementary Material 1.

## Data Availability

The datasets used and/or analyzed during the current study are available from the corresponding author on reasonable request.

## Abbreviations

TSP50: Testes-specific protease 50
(CSC)-like: Cancer stem cell-like
EMT: Epithelial-mesenchymal transition
ALDH: Aldehyde dehydrogenase
HE: Haematoxylin–eosin
IHC: Immunohistochemistry
EGF: Epidermal growth factor
bFGF: Basic fibroblast growth factor
ELDA: Extreme limiting dilution analysis
OD: Optical density
BCSC: Breast cancer stem cell
OS: Overall survival
DMFS: Distant metastasis free survival
ROC: Receiver operating characteristic curves
PFS: Progression free survival
NC: Negative control
IF: Immunofluorescence
PI3P: Phosphatidylinositol 3-phosphate
PI2P: Phosphatidylinositol 4,5-biphosphate
Co-IP: Co-immunoprecipitation
p85BD: P85 binding domain
RBD: Ras-binding domain
IP: Immunoprecipitation
ESC: Embryonic stem cell
MMPs: Matrix metalloproteinases
RTKs: Receptor tyrosine kinases
