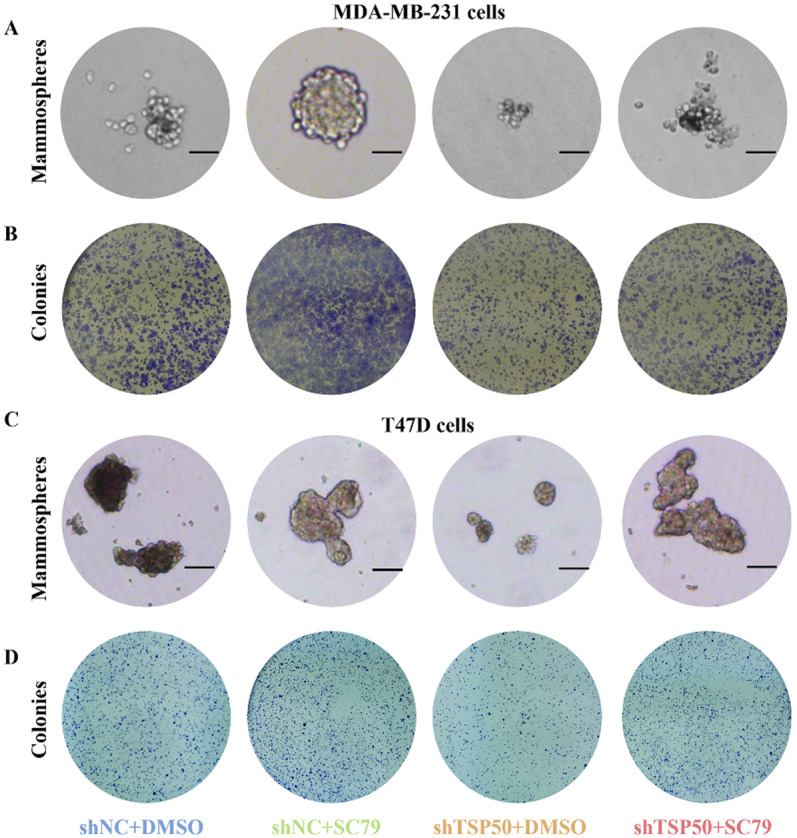# Correction: TSP50 facilitates breast cancer stem cell‑like properties maintenance and epithelial‑mesenchymal transition via PI3K p110α mediated activation of AKT signaling pathway

**DOI:** 10.1186/s13046-026-03760-0

**Published:** 2026-06-18

**Authors:** Feng Gao, Sichen Liu, Jing Wang, Gang Wei, Chunlei Yu, Lihua Zheng, Luguo Sun, Guannan Wang, Ying Sun, Yongli Bao, Zhenbo Song

**Affiliations:** 1 National Engineering Laboratory for Druggable Gene and Protein Screening, Northeast Normal University, NO.5268 Renmin Street, Changchun, 130117 China; 2 NMPA Key Laboratory for Quality Control of Cell and Gene Therapy Medicine Products, Northeast Normal University, NO.5268 Renmin Street, Changchun, 130117 China; 3 China International Joint Research Center for Human Stem Cell Bank, Northeast Normal University, Changchun, Jilin 130024 China; 4 Department of Neurosurgery/Neuro‑Oncology, SunYat-Sen University Cancer Center, State Key Laboratory of Oncology in South China, Collaborative Innovation Center for Cancer Medicine, Guangzhou, Guangdong 510060 China; 5 Department of Breast Surgery, Jilin Province Cancer Hospital, Changchun, 130012 China

**Correction: J Exp Clin Cancer Res 43 (1), 201 (2024)**

**https://doi.org/10.1186/s13046-024-03118-4**

Following the publication of the original article [1], the authors identified errors in Fig. S11, specifically:

- Figure S11D - The clone formation image used for the T47D cells is incorrect.

These errors were caused by unintentionally covering the correct image during figure preparation. The corrected figures are provided below:

The corrections do not compromise the validity of the conclusions and the overall content of the article. The original article [1] has been corrected.

**Incorrect figure S11**.

**Fig. S11:**
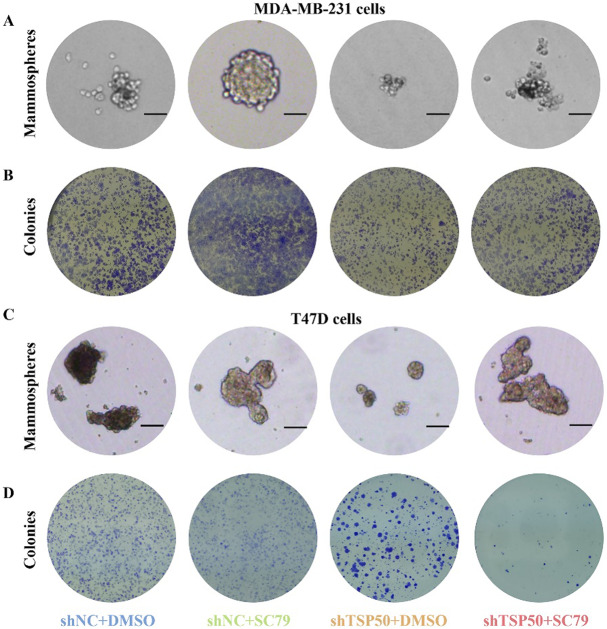
Representative images of mammospheres and colonies**. A-B** Representative images of mammospheres and colonies for SC-79-treated MDA-MB-231 cells with TSP50 knockdown. **C-D** Representative images of mammospheres and colonies for SC-79-treated T47D cells with TSP50 knockdown. Scale bar, 25 μm

**Correct figure S11**.

**Fig. S11 **Representative images of mammospheres and colonies.** (A-B)** Representative images of mammospheres and colonies for SC-79-treated MDA-MB-231 cells with TSP50 knockdown. **(C-D)** Representative images of mammospheres and colonies for SC-79-treated T47D cells with TSP50 knockdown. Scale bar, 25 μm

**Figure:**