# Supplementary material for: TSP50 facilitates breast cancer stem cell-like properties maintenance and epithelial-mesenchymal transition via PI3K p110α mediated activation of AKT signaling pathway
Source: J Exp Clin Cancer Res. 2024 Jul 20;43:201. doi: 10.1186/s13046-024-03118-4 (PMC11264956; doi:10.1186/s13046-024-03118-4)
Supplement: Supplementary file 1 — Supplementary Material 1. [file 13046_2024_3118_MOESM1_ESM.docx]

**Supplementary Materials for**

**TSP50 facilitates breast cancer stem cell-like properties maintenance and epithelial-mesenchymal transition via PI3K p110α mediated activation of AKT signaling pathway**

*Corresponding author: Zhenbo Song, songzb484@nenu.edu.cn；Yongli Bao, baoyl800@nenu.edu.cn.

**This PDF file includes:**

Fig. S1 to S13

Key Resources Table

**Supplementary Figures**

**Fig. S1**


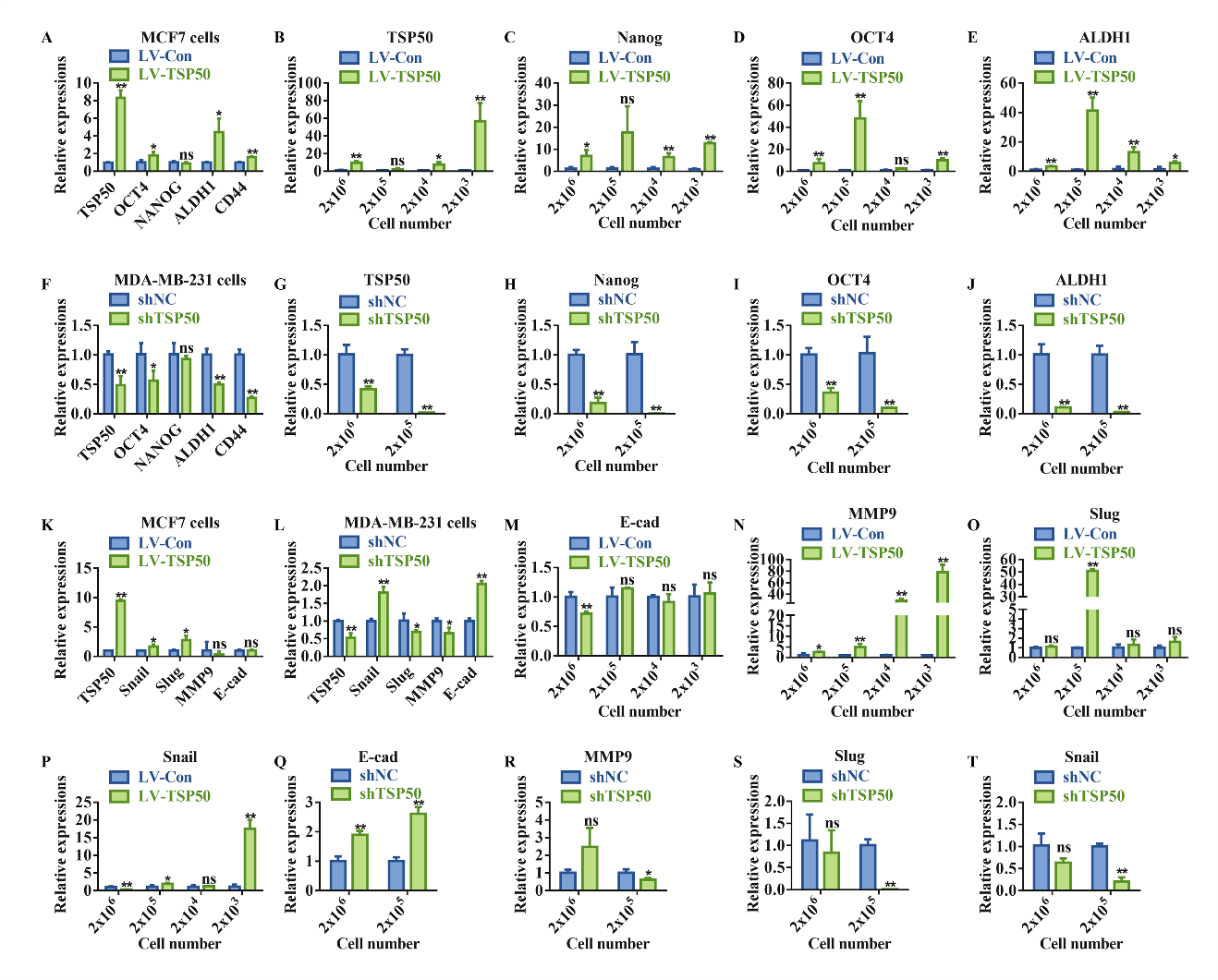


**Fig. S1** **TSP50 increased the mRNA expression levels of BCSC-/EMT-related markers. (A)** The mRNA expression levels detection results of BCSC-related markers in MCF7 cells stably overexpressing NC or TSP50. **(B-E)** Stable TSP50 or NC overexpressed mammospheres were collected for limited dilution and tumorigenesis assay. The mRNA levels of BCSC-related markers were detected. **(F)** The mRNA expression levels detection results of BCSC-related markers in MDA-MB-231 cells transfected with TSP50 shRNA (shTSP50) and control (shNC). **(G-J)** Stable TSP50-knockdown and shNC mammospheres were collected for limited dilution and tumorigenesis assay. The BCSC-related markers mRNA levels were detected. **(K-L)** The mRNA expression levels detection results of EMT-related markers in MCF7 cells after TSP50 overexpression or knockdown. **(M-P)** Stable overexpression of TSP50 or NC mammospheres were seeded into nude mice. The mRNA expression levels of EMT-related markers were determined by qRT-PCR. **(Q-T)** Stable TSP50-knockdown or shNC mammospheres were seeded into nude mice. The mRNA expression levels of EMT-related markers were detected by qRT-PCR. N=3 biologically independent replicates for *in vitro* and *in* *vivo* qRT-PCR assay (2 × 10^3^-10^5^ MCF7 cell groups and 2 × 10^5^ MDA-MB-231 cell groups). N=2 biologically independent replicates for *in* *vivo* qRT-PCR assay (2 × 10^6^ cell groups). Student’s t-test statistical analysis was used. *^*^p < 0.05*, ***p < 0.01* and ns, no significance.

**Fig. S2**


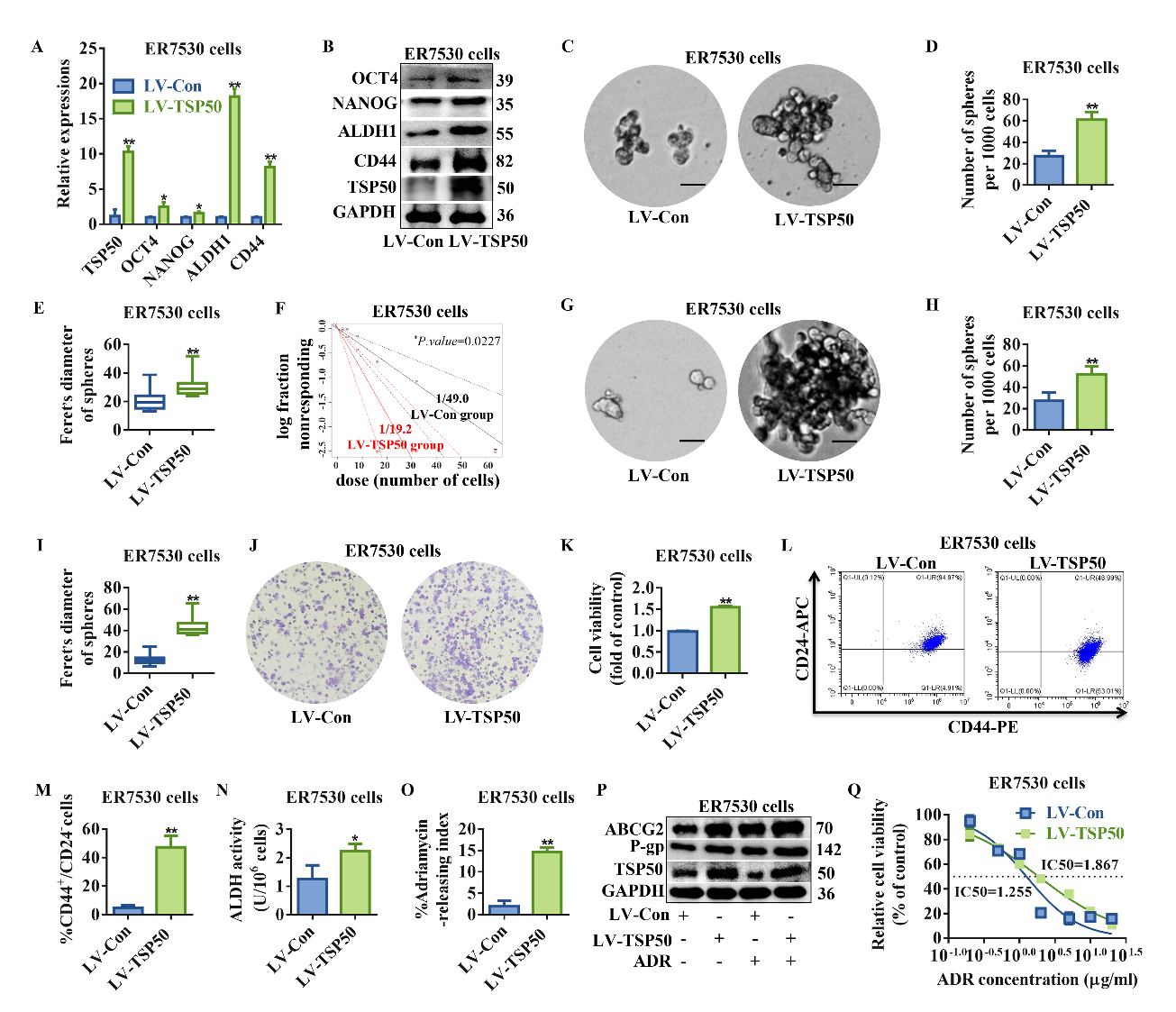


**Fig. S2 Overexpression of TSP50 increases CSC-like phenotypes in ER7530 cells.** **(A-B)** The mRNA and protein expression levels of BCSC-related markers OCT4, NANOG, ALDH1 and CD44 in ER7530 cells stably overexpressing NC or TSP50. **(C-E)** Representative spheroid images derived from the NC or TSP50-overexpressed ER7530 cells (C). Scale bar, 25 μm. Primary mammosphere number (D) and size (E) were calculated. **(F)** The linear regression plot generated by ELDA for the *in vitro* limiting dilution assay with NC or TSP50 transfected ER7530 cells. **(G-I)** Representative spheroid images derived from the NC or TSP50-overexpressed mammospheres (G). Scale bar, 25 μm. Secondary mammosphers number (H) and size (I) were calculated. **(J-K)** Representative colony formation images derived from the stable TSP50 or NC overexpressed ER7530 cells and the number of colonies analysis results. **(L-M)** The CD44^+^/CD24^-^ cell subpopulation proportion analysis results by flow cytometry. **(N)** ALDH activity detection results. **(O-Q)** The ADR pumping rate (O), expression levels of drug resistance proteins ABCG2 and P-gp (P) and IC50 values of ADR (Q) detection results in ER7530 cells stably overexpressing NC or TSP50. N=3 biologically independent replicates. *^*^p < 0.05* and ***p < 0.01*, as compared with NC group by Student’s t-test.

**Fig. S3**


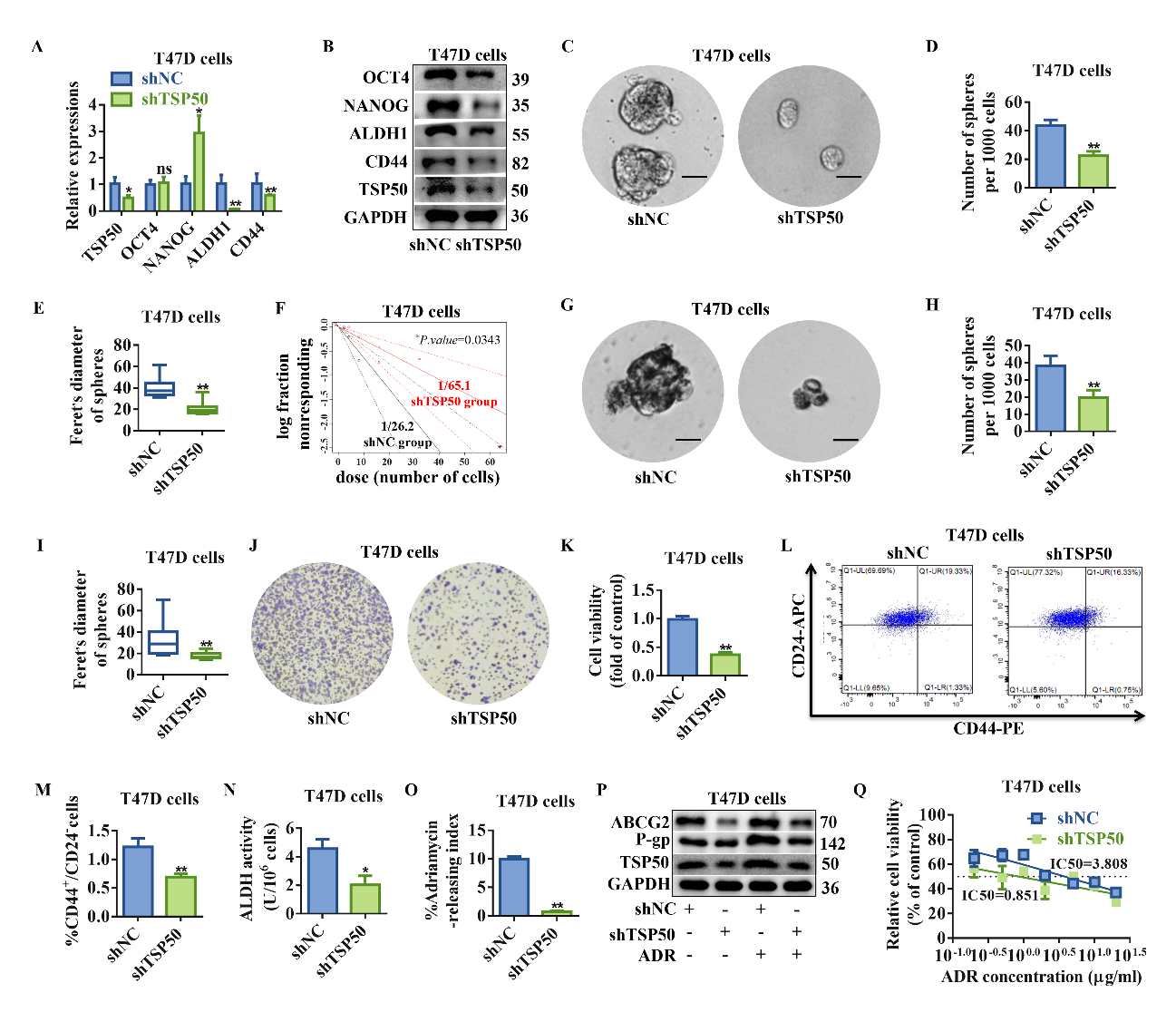


**Fig. S3 Inhibition of TSP50 attenuates CSC-like phenotypes in T47D cells. (A-B)** The mRNA and protein expression levels of BCSC-related markers OCT4, NANOG, ALDH1 and CD44 in T47D cells transfected with TSP50 shRNA (shTSP50) and the control (shNC). **(C-E)** Representative spheroid images derived from the T47D cells transfected with shNC or shTSP50 (C). Scale bar, 25 μm. Primary mammosphere number (D) and size (E) were calculated. **(F)** The linear regression plot generated by ELDA for the *in vitro* limiting dilution assay with T47D-shNC or T47D-shTSP50 cells. **(G-I)** Representative images of spheroids derived from the mammospheres transfected with shNC or shTSP50 (G). Scale bar, 25 μm. Secondary mammosphere number (H) and size (I) were calculated. **(J-K)** Representative colony formation images derived from the shNC or shTSP50 transfected T47D cells and the number of colonies analysis results. **(L-M)** The CD44^+^/CD24^-^ cells subpopulation proportion analysis results by flow cytometry. **(N)** ALDH activity detection results. **(O-Q)** The ADR pumping rate (O), expression levels of drug resistance proteins ABCG2 and P-gp (P) and IC50 values of ADR (Q) detection results in shNC or shTSP50 transfected T47D cells. N=3 biologically independent replicates. *^*^p < 0.05* and ***p < 0.01*, as compared with shNC group by Student’s t-test, ns, no significance.

**Fig. S4**


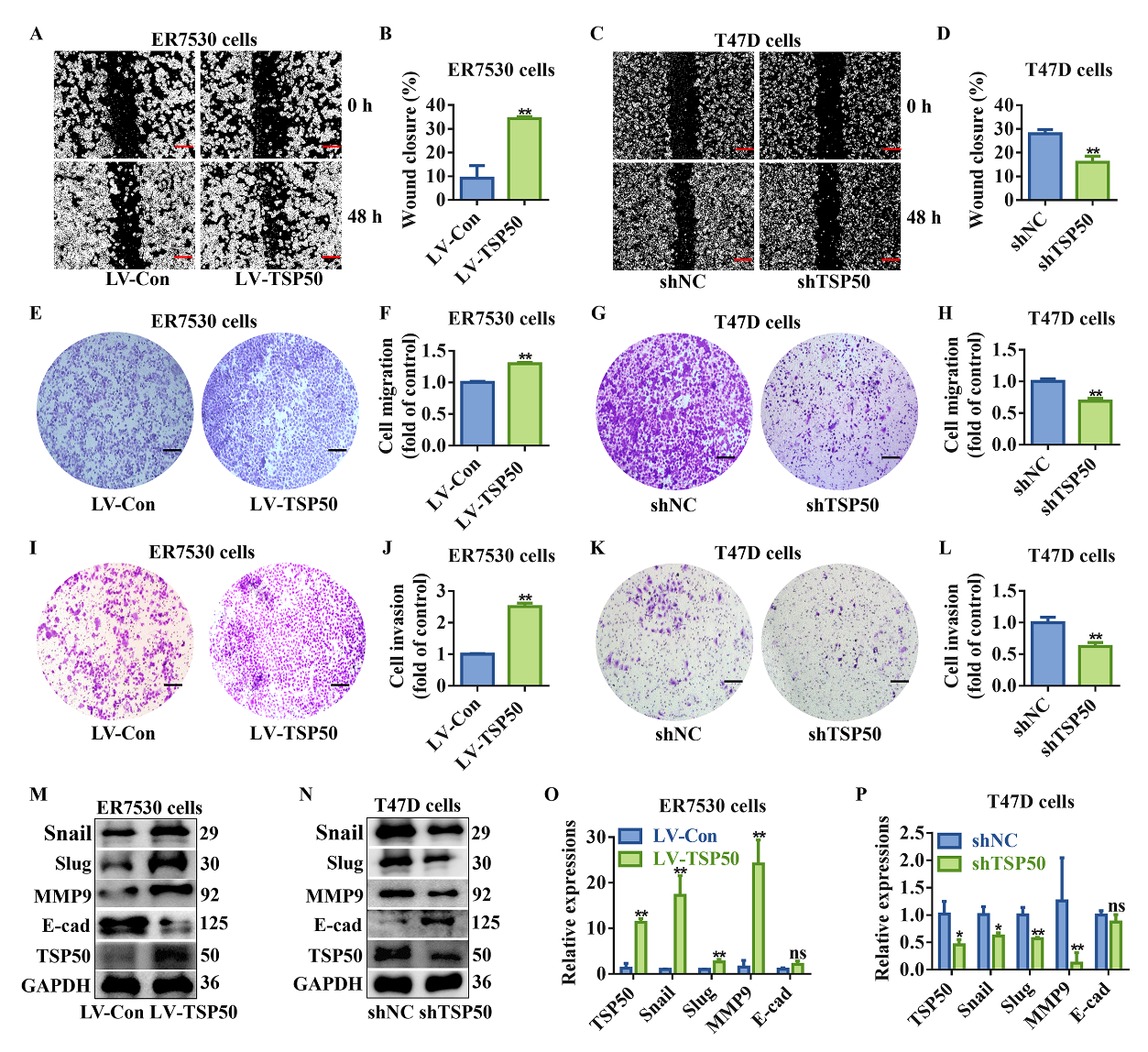


**Fig. S4 TSP50 is involved in the regulation of breast cancer cell metastasis and EMT. (A-B)** Wound healing of ER7530 cells stably overexpressing NC or TSP50. **(C-D)** Wound healing of T47D cells transfected with shNC or shTSP50. **(E-F)** Migration of ER7530 cells stably overexpressing NC or TSP50. **(G-H)** Migration of T47D cells transfected with shNC or shTSP50. **(I-J)** Invasion of ER7530 cells stably overexpressing NC or TSP50. **(K-L)** Invasion of T47D cells transfected with shNC or shTSP50. **(M-N)** The protein expression levels of EMT-related markers E-cad, MMP9, Slug and Snail in ER7530 and T47D cells. **(O-P)** The mRNA expression levels of EMT-related markers E-cad, MMP9, Slug and Snail in ER7530 and T47D cells. Scale bar, 25 μm. N=3 biologically independent replicates. Student’s t-test statistical analysis was used. *^*^p < 0.05*, ***p < 0.01* and ns, no significance.

**Fig. S5**


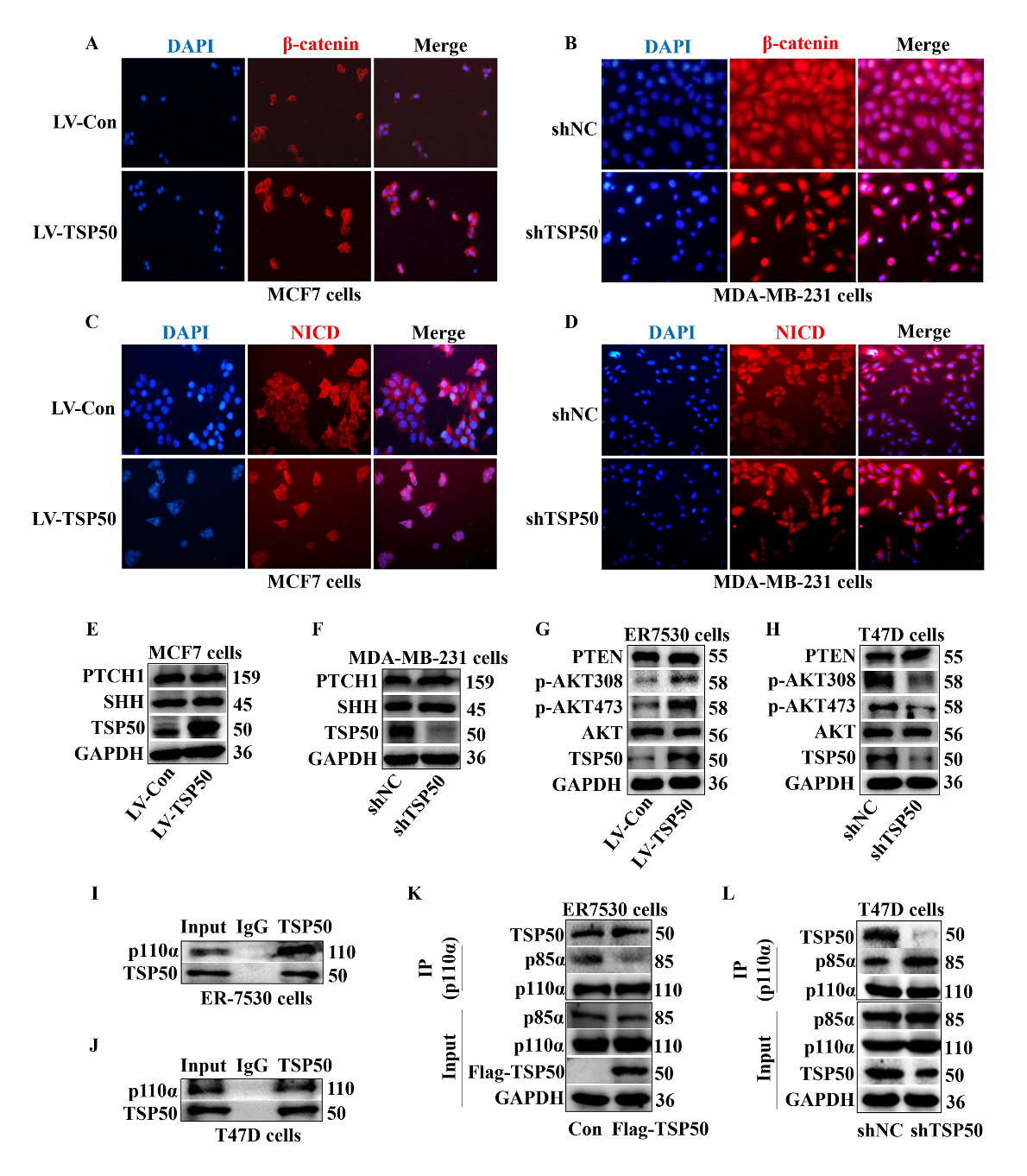


**Fig. S5 The PI3K/AKT signaling pathway is activated by TSP50. (A-B)** The effect of TSP50 overexpression or knockdown on nuclear translocation of β-catenin by IF assay. **(C-D)** The effect of TSP50 overexpression or knockdown on nuclear translocation of NICD by IF assay. **(E-F)** The effect of TSP50 overexpression or knockdown on PTCH1 and SHH protein expression levels by Western blot. **(G)** Western blot detection results of PI3K/AKT signaling-related marker expression levels in ER7530 cells stably overexpressing NC or TSP50. **(H)** Western blot detection results of PI3K/AKT signaling-related marker expression levels in T47D cells transfected with shNC or shTSP50. **(I-J)** ER7530 and T47D cells were harvested and subjected to Co-IP with anti- TSP50 antibody, followed by Western blot analysis with anti-p110α antibody. **(K-L)** ER7530 cells stably overexpressing NC or TSP50 and T47D cells transfected with shNC or shTSP50 were harvested and subjected to Co-IP with anti-p110α antibody, followed by Western blot analysis with anti-p85α and anti-TSP50 antibodies. N=3 biologically independent replicates.

**Fig. S6**


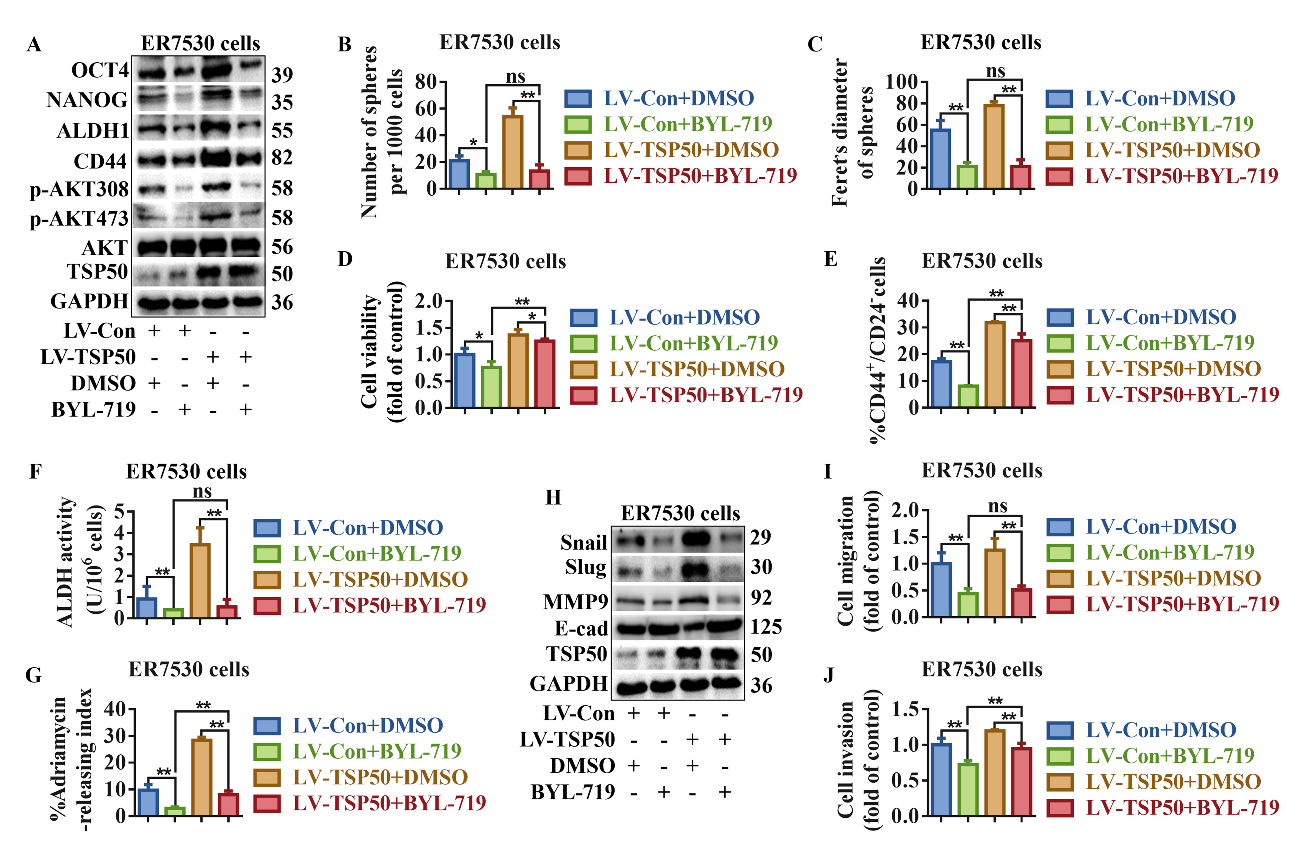


**Fig. S6 BYL-719 attenuates CSC-like phenotypes maintenance, EMT and cell metastasis promotion effects of TSP50 in ER7530 cells.** TSP50 or NC stably overexpressed ER7530 cells were treated with BYL-719. **(A)** The levels of indicated BCSC-related markers were determined by Western blot. **(B-C)** Primary mammosphere number and size were calculated. **(D)** The number of colonies analysis results. **(E)** The subpopulation proportion analysis results of CD44^+^/CD24^-^ cells. **(F)** ALDH activity detection results. **(G)** The ADR pumping rate. **(H)** The levels of EMT-related markers were determined by Western blot. **(I)** Migration assay detection results. **(J)** Invasion assay detection results. N=3 biologically independent replicates. Student’s t-test or one-way ANOVA statistical analysis was used. *^*^p < 0.05*, ***p < 0.01* and ns, no significance.

**Fig. S7**


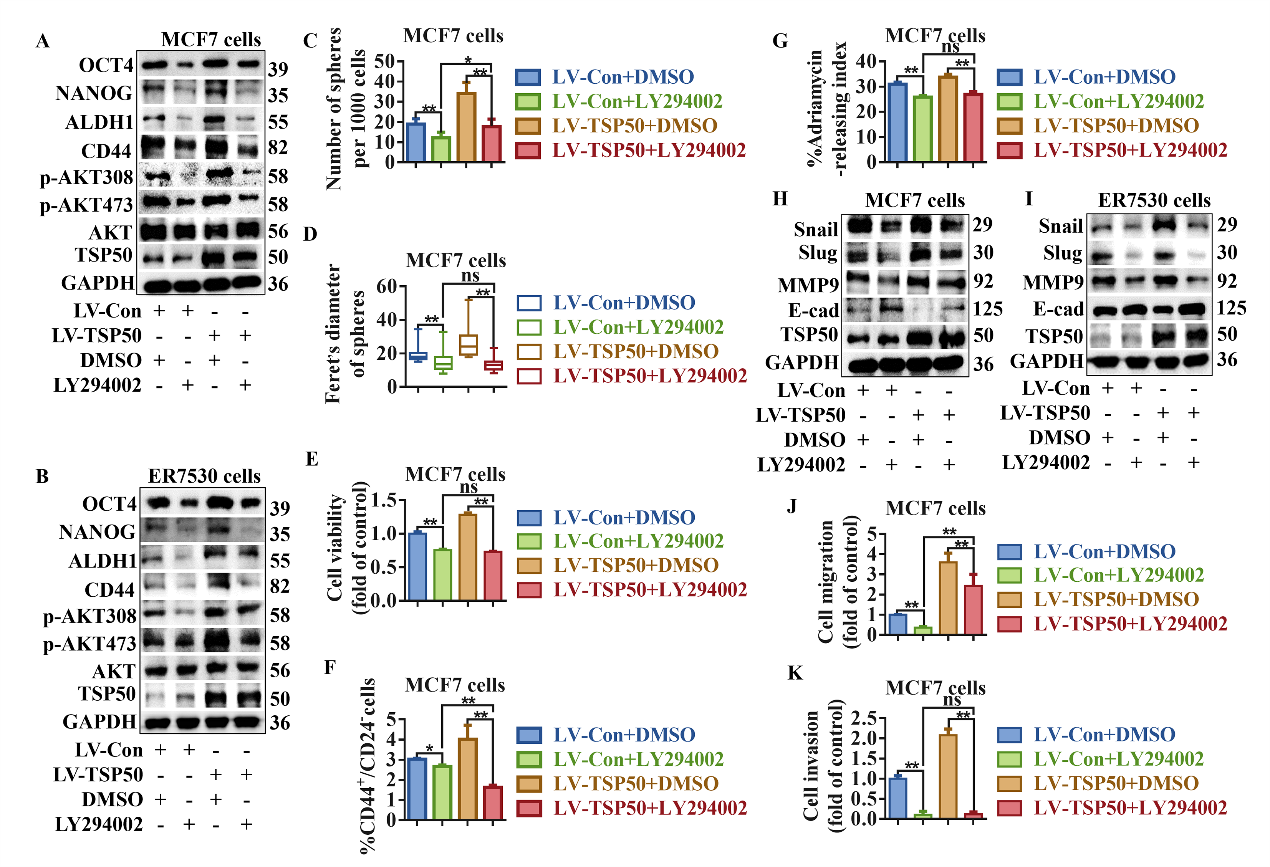


**Fig. S7 LY294002 partially reverses the CSC-like and EMT phenotypes of TSP50-overexpressed breast cancer cells.** TSP50 or NC stably overexpressed MCF7 and ER7530 cells were treated with LY294002. **(A-B)** The levels of the indicated BCSC-related markers were determined by Western blot. **(C-D)** Primary mammosphere number and size in MCF7 cell groups were calculated. **(E)** The subpopulation proportion analysis results of CD44^+^/CD24^-^ MCF7 cells. **(F)** The ADR pumping rate in MCF7 cell groups. **(G)** The number of colonies analysis results in MCF7 cell groups. **(H-I)** The levels of EMT-related markers were determined by Western blot. **(J)** MCF7 cell migration detection results. **(K)** MCF7 cell invasion detection results. N=3 biologically independent replicates. Student’s t-test or one-way ANOVA statistical analysis was used. *^*^p < 0.05*, ***p < 0.01* and ns, no significance.

**Fig. S8**


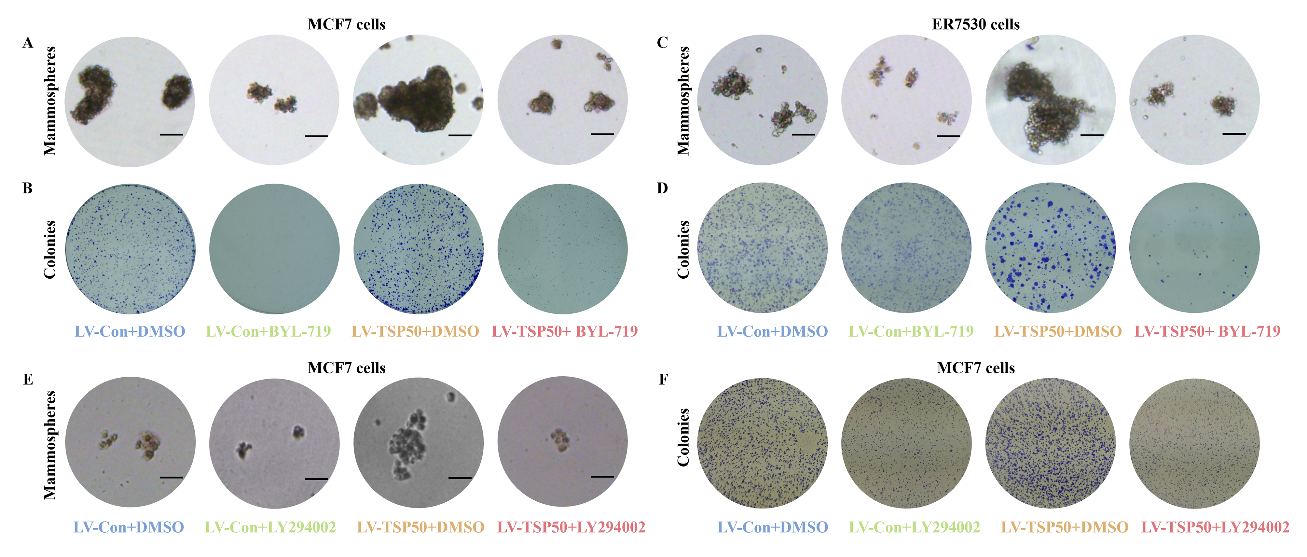


**Fig. S8** **Representative images of mammospheres and colonies.** (**A-B**) Representative images of mammospheres and colonies for TSP50-overexpressed MCF7 cells treated with BYL-719. (**C-D**) Representative images of mammospheres and colonies for TSP50-overexpressed ER7530 cells treated with BYL-719. (**E-F**) Representative images of mammospheres and colonies for TSP50-overexpressed MCF7 cells treated with LY294002. Scale bar, 25 μm.

**Fig. S9**


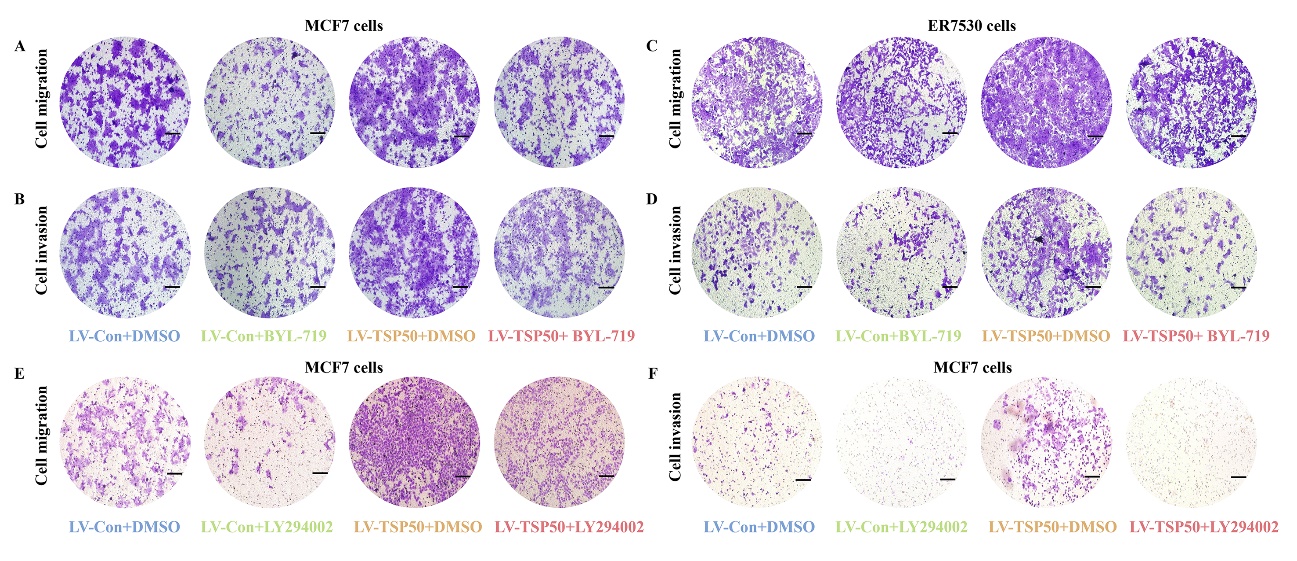


**Fig. S9** **Representative images of** **cell migration and invasion. (A-B)** Representative images of cell migration and invasion for TSP50-overexpressed MCF7 cells treated with BYL-719. **(C-D)** Representative images of cell migration and invasion for TSP50-overexpressed ER7530 cells treated with BYL-719. **(E-F)** Representative images of cell migration and invasion for TSP50-overexpressed MCF7 cells treated with LY294002. Scale bar, 25 μm.

**Fig. S10**


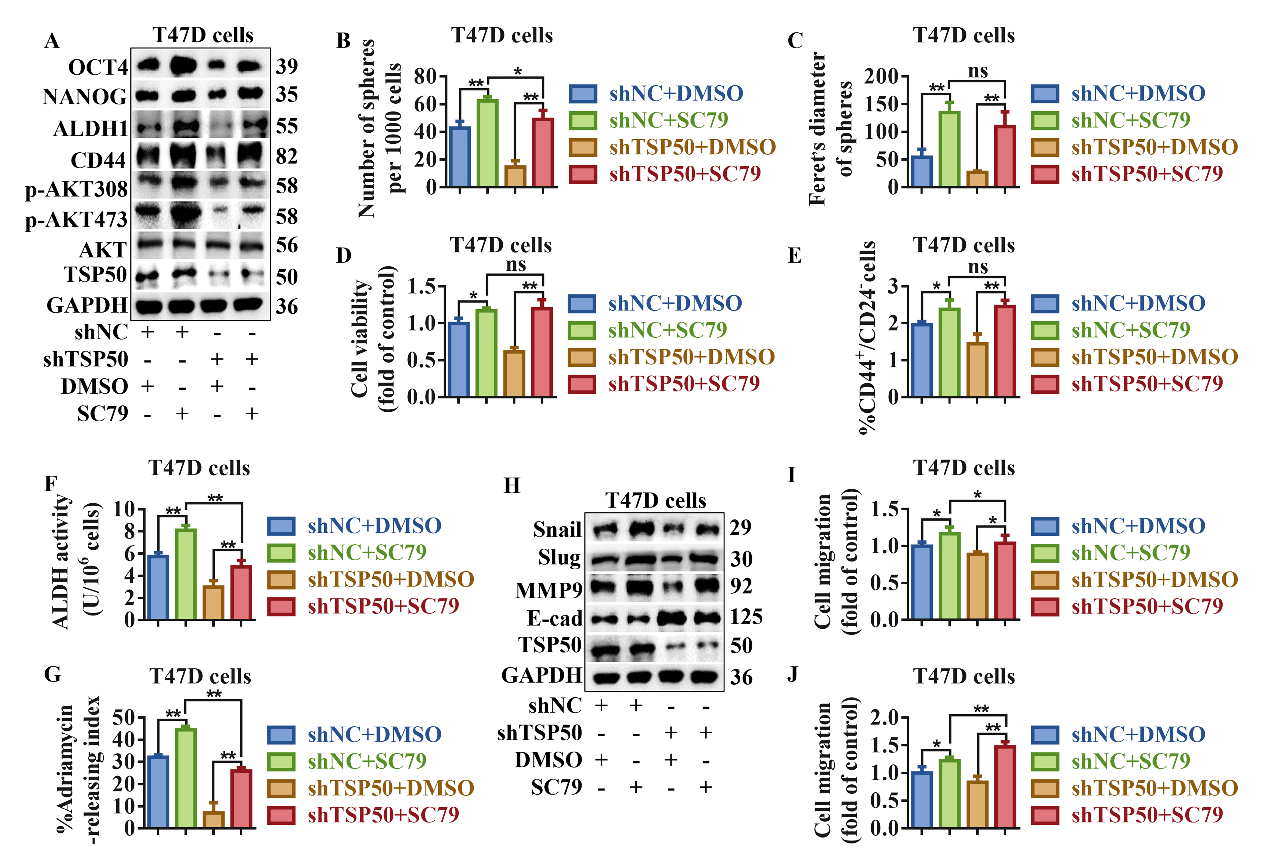


**Fig. S10 SC79 partially restores CSC-like and EMT phenotypes in T47D cells with TSP50 knockdown.** The shNC or shTSP50 transfected T47D cells were treated with SC79. **(A)** The levels of indicated BCSC-related markers were determined by Western blot. **(B-C)** Primary mammospheres number and size were calculated. **(D)** The number of colonies analysis results. **(E)** The subpopulation proportion analysis results of CD44^+^/CD24^-^ cells. **(F)** ALDH activity detection results. **(G)** The ADR pumping rate. **(H)** The levels of EMT-related markers were determined by Western blot. **(I)** Migration assay detection results. **(J)** Invasion assay detection results. N=3 biologically independent replicates. Student’s t-test or one-way ANOVA statistical analysis was used. *^*^p < 0.05*, ***p < 0.01* and ns, no significance.

**Fig. S11**


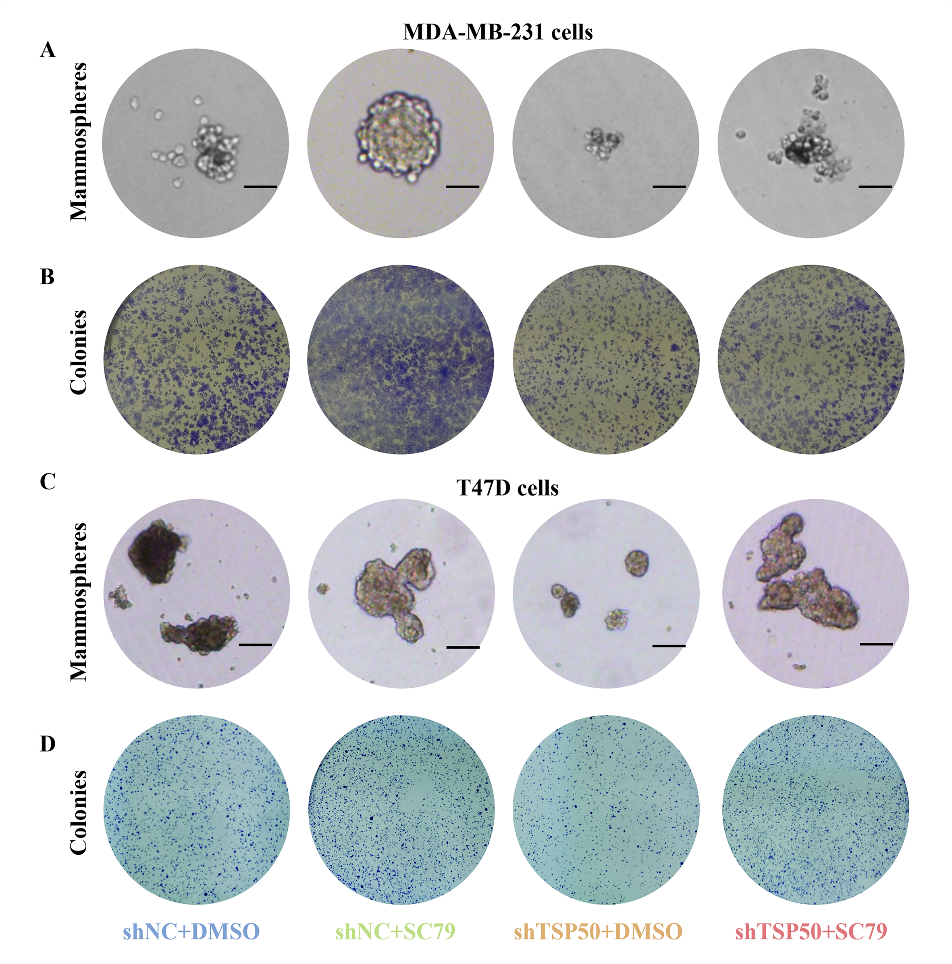


**Fig. S11 Representative images of mammospheres and colonies. (A-B)** Representative images of mammospheres and colonies for SC-79-treated MDA-MB-231 cells with TSP50 knockdown. **(C-D)** Representative images of mammospheres and colonies for SC-79-treated T47D cells with TSP50 knockdown. Scale bar, 25 μm.

**Fig. S12**


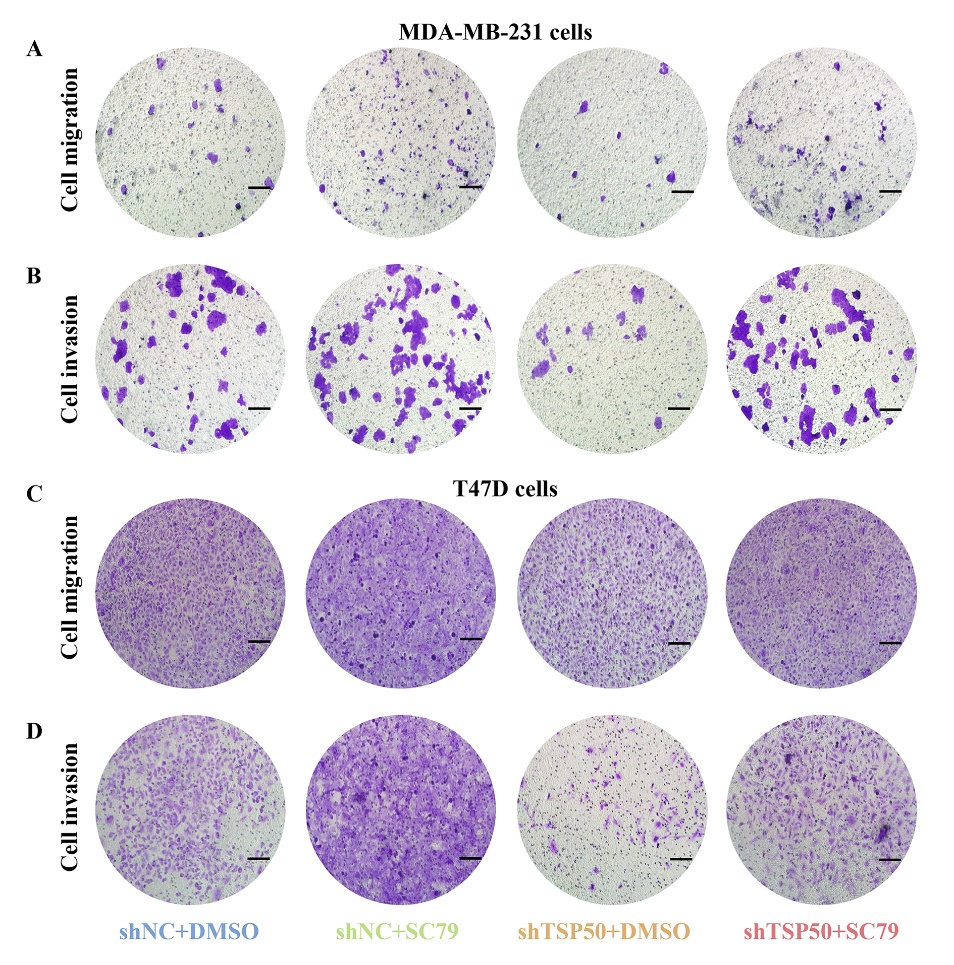


**Fig. S12 Representative images of cell migration and invasion. (A-B)** Representative images of cell migration and invasion for SC-79-treated MDA-MB-231 cells with TSP50 knockdown. **(C-D)** Representative images of cell migration and invasion for SC-79-treated T47D cells with TSP50 knockdown. Scale bar, 25 μm.

**Fig. S13**


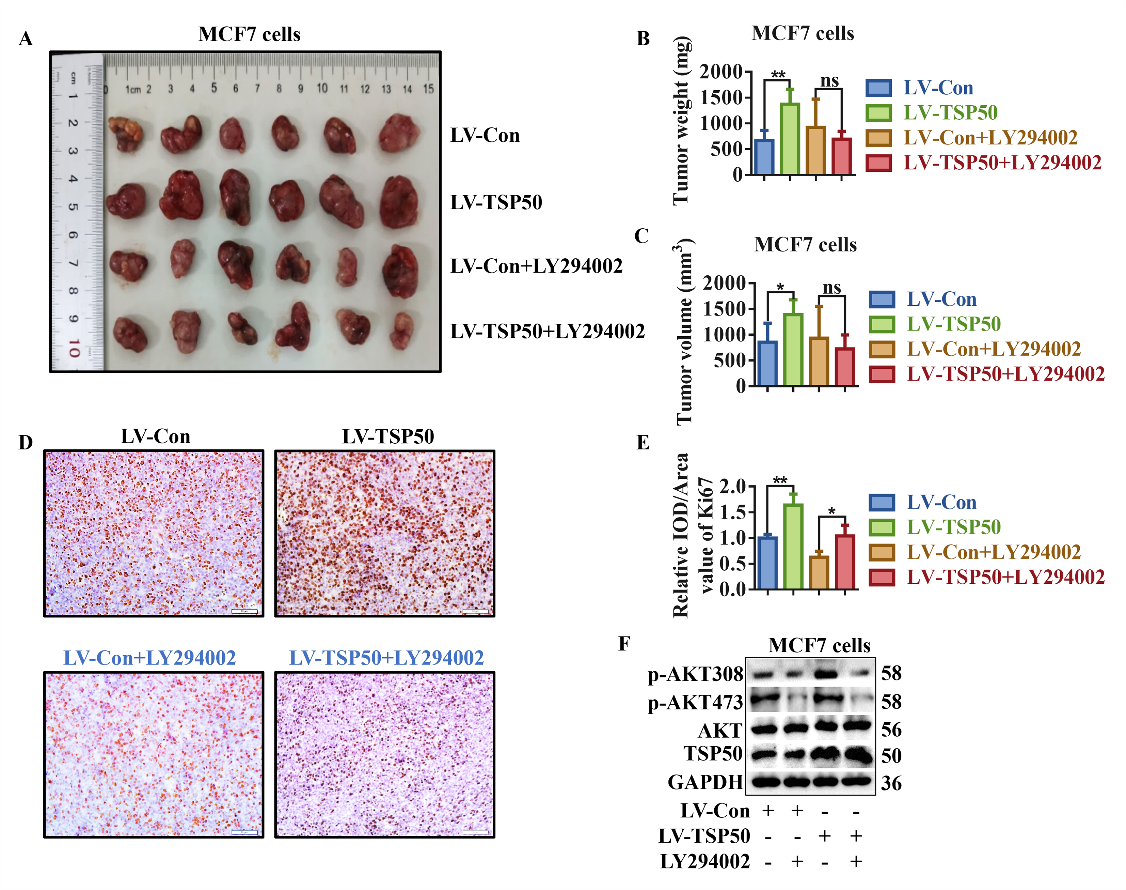


**Fig. S13** **LY294002 weakens TSP50 tumor-promoting effects *in vivo*.** The nude mice were injected with NC or TSP50 stably overexpressing mammospheres and then treated with LY294002. **(A)** Photograph of the xenograft tumor. **(B)** Tumor weight. **(C)** Tumor volume. **(D-E)** Results of the IHC assay for Ki67 levels. **(F)** PI3K/AKT signal-related markers Western blot detection results. Scale bar, 50 μm. N=3 biologically independent replicates. Student’s t-test statistical analysis was used. *^*^p < 0.05*, ***p < 0.01* and ns, no significance.

**Key Resources Table**

| REAGENT or RESOURCE | | SOURCE | IDENTIFIER |
| --- | --- | --- | --- |
| **Antibodies** | |  |  |
| TSP50  TSP50 | | Lab-made  Proteintech, Wuhan, China | N/A  12574-1-AP |
| CD44 | | Proteintech, Wuhan, China | 15675-1-AP |
| ALDH1 | | Proteintech, Wuhan, China | 15910-1-AP |
| NANOG | | Proteintech, Wuhan, China | 14295-1-AP |
| OCT4 | | Proteintech, Wuhan, China | 11263-1-AP |
| E-cad | | Proteintech, Wuhan, China | 20874-1-AP |
| MMP9 | | Proteintech, Wuhan, China | 10375-2-AP |
| Slug | | Proteintech, Wuhan, China | 12129-1-AP |
| Snail | | Proteintech, Wuhan, China | 13099-1-AP |
| P-gp | | Proteintech, Wuhan, China | 22336-1-AP |
| ABCG2 | | Wanleibio, Shenyang, China | WL03192 |
| GAPDH | | Proteintech, Wuhan, China | 60004-1-Ig |
| AKT | | Proteintech, Wuhan, China | 10176-2-AP |
| p-AKT473 | | Proteintech, Wuhan, China | 66444-1-Ig |
| p-AKT308 | | Proteintech, Wuhan, China | 29163-1-AP |
| PTEN | | Proteintech, Wuhan, China | 22034-1-AP |
| PI3K p110α | | Proteintech, Wuhan, China | 67071-1-Ig |
| PI3K p110α | | Santa Cruz, Dallas, USA | sc-293172 |
| PI3K p85α  Ki67 | | Proteintech, Wuhan, China  Proteintech, Wuhan, China | 60225-1-Ig  27309-1-AP |
| Flag | | Proteintech, Wuhan, China | 20543-1-AP |
| Myc | | Proteintech, Wuhan, China | 60003-2-Ig |
| β-catenin | | Proteintech, Wuhan, China | 51067-2-AP |
| NICD | | Proteintech, Wuhan, China | 20687-1-AP |
| SHH | | Wanleibio, Shenyang, China | WL01472 |
| PTCH1 | | Wanleibio, Shenyang, China | WL04030 |
| CD44-PE | | BioLegend, SanDiego, USA | 103007 |
| CD24-APC | | BioLegend, SanDiego, USA | 311117 |
| Mouse IgG | | Santa Cruz, Dallas, USA | sc-2025 |
| Rabbit IgG | | Proteintech, Wuhan, China | 66467-1-Ig |
| IPKine™ HRP, Goat Anti-Mouse IgG LCS | | Abbkine, Wuhan, China | A25012 |
| Mouse IgG (H+L) HRP | | Affinity, USA | S0002 |
| Rabbit IgG (H+L) HRP | | Affinity, USA | S0001 |
| FITC-labeled Mouse IgG (H+L) | | Beyotime, Shanghai, China | A0562 |
| Cy3-labeled Rabbit IgG (H+L) | | Beyotime, Shanghai, China | A0521 |
| **Critical Reagent, cell culture supplies and commercial assays** | | | |
| RPMI 1640 medium | Corning, New York, USA | | 10-040-CV |
| DMEM/F-12 (1:1) medium | GIBCO, New York, USA | | 12400-024 |
| EGF | Peprotech, New Jersey, USA | | GMP100-15 |
| bFGF | Peprotech, New Jersey, USA | | 100-18B |
| FBS | Hyclone, Australia | | SH30084.03 |
| Penicillin-streptomycin | Hyclone, Australia | | SV30010 |
| Trypsin | Beyotime,Shanghai,China | | C0201 |
| Trizol | Invitrogen, California, USA | | 15596026CN |
| First-Strand cDNA Synthesis SuperMix | TransGen Biotech, Beijing, China | | AF301-02 |
| qPCR SuperMix | TransGen Biotech, Beijing, China | | AQ101-02 |
| RIPA buffer | Boster, Wuhan, China | | AR0102 |
| ECL luminous fluid | Boster, Wuhan, China | | AR1191 |
| DAPI | Beyotime, Shanghai, China | | C1002 |
| FuGENE HD Transfection Reagent | Roche, Basel, Switzerland | | 04709705001 |
| PVDF membrane | Sigmaaldrich, Missouri, USA | | 03010040001 |
| Protein A/G Magnetic Beads | MCE, New Jersey, USA | | HY-K0202 |
| PI3K elisa kit  ALDH test kit | Yanke Biotech, Huangshi, China  Solarbio, Beijing, China | | N/A  BC0755 |
| Adriamycin | Beyotime, Shanghai, China | | ST1285 |
| DMSO | Sangon Biotech, Shanghai, China | | A600163 |
| Matrigel  Polybrene  Puromycin  SC79  LY294002  BYL-79  Cell Plasma Membrane Staining Kit with DiD  Plasmid extraction kit  Rabbit two-step detection kit  Mouse two-step detection kit  Ultra-low attachment plates | Corning, New York, USA  Beyotime, Shanghai, China  Beyotime, Shanghai, China  MCE, New Jersey, USA  MCE, New Jersey, USA  MCE, New Jersey, USA  Beyotime, Shanghai, China  Tiangen Biotech, Beijing, China  Zhongshan Jinqiao Biotech, Beijing, China  Zhongshan Jinqiao Biotech, Beijing, China  Jindian Biotech, Qingdao, China | | 354234  C0351  ST551  HY-18749  HY-10108  HY-15244  C1995S  DP117  PV-6001  PV-6002  16106-2SULH |
| Transwell | Corning, New York, USA | | YM-2013A |
| **Expression Plasmids** |  | |  |
| shTSP50s | Sangon Biotech, Shanghai, China | | N/A |
| pcDNA3.1-TSP50 | Lab-made | | N/A |
| pCMV-TSP50-3×flag  pCMV-TSP50-H153A-3×flag | Miaoling Bio., Wuhan, China  Miaoling Bio., Wuhan, China | | N/A  N/A |
| pCMV-TSP50-D206A-3×flag | Miaoling Bio., Wuhan, China | | N/A |
| pCMV-TSP50-T310A-3×flag | Miaoling Bio., Wuhan, China | | N/A |
| pCMV-p110α-3×Myc | Miaoling Bio., Wuhan, China | | N/A |
| pCMV-p85BD-3×Myc | Miaoling Bio., Wuhan, China | | N/A |
| pCMV-RBD-3×Myc | Miaoling Bio., Wuhan, China | | N/A |
| pCMV-C2-3×Myc  pCMV-Helical-3×Myc | Miaoling Bio., Wuhan, China  Miaoling Bio., Wuhan, China | | N/A  N/A |
| pCMV-Catalytic-3×Myc | Miaoling Bio., Wuhan,China | | N/A |
| Plv3-TSP50 | Miaoling Bio., Wuhan,China | | N/A |
| Plv3-shTSP50 | Miaoling Bio., Wuhan, China | | N/A |
| **Cell Lines** |  | |  |
| MDA-MB-231 cells | Chinese Academy of Sciences,China | | N/A |
| MCF7 cells | Chinese Academy of Sciences,China | | N/A |
| T47D cells | Chinese Academy of Sciences,China | | N/A |
| ER7530 cells | Chinese Academy of Sciences,China | | N/A |
| SKBR3 cells | Chinese Academy of Sciences,China | | N/A |
| **Software and Algorithms** |  | |  |
| IBM SPSS Statistics  Image J | IBM software  Image J software | | N/A  N/A |
| GraphPad Prism 6.0 | GraphPad software | | N/A |
| TISIDB | TISIDB online database | | http://cis.hku.hk/TISIDB/index.php |
| KM plotter | KM plotter online database | | http://kmplot.com/ |
| ROC plotter | ROC plotter online database | | www.rocplot.org |
| GEPIA2 | Gene Expression Profiling Interactive Analysis database | | http://gepia2.cancer-pku.cn/#index |
| ELDA | Extreme Limiting Dilution Analysis online software | | http://bioinf.wehi.edu.au/software/elda |
